# Small-Angle X-ray Scattering (SAXS) Combined with SAXS-Driven Molecular Dynamics for Structural Analysis of Multistranded RNA Assemblies

**DOI:** 10.1021/acsami.4c12397

**Published:** 2024-11-27

**Authors:** Lewis A. Rolband, Kriti Chopra, Leyla Danai, Damian Beasock, Hubertus J.J. van Dam, Joanna K. Krueger, James Byrnes, Kirill A. Afonin

**Affiliations:** † Nanoscale Science Program, Department of Chemistry, University of North Carolina Charlotte, Charlotte, North Carolina 28223, United States; ‡ Computational Science Initiative, Brookhaven National Laboratory, Upton, New York 11973, United States; § Condensed Matter Physics and Materials Science Dept, Brookhaven National Laboratory, Upton, New York 11973, United States; ∥ National Synchrotron Light Source II, Brookhaven National Laboratory, Upton, New York 11973, United States

**Keywords:** SAXS, MD simulations, NANPs, RNA self-assembly, RNA nanotechnology

## Abstract

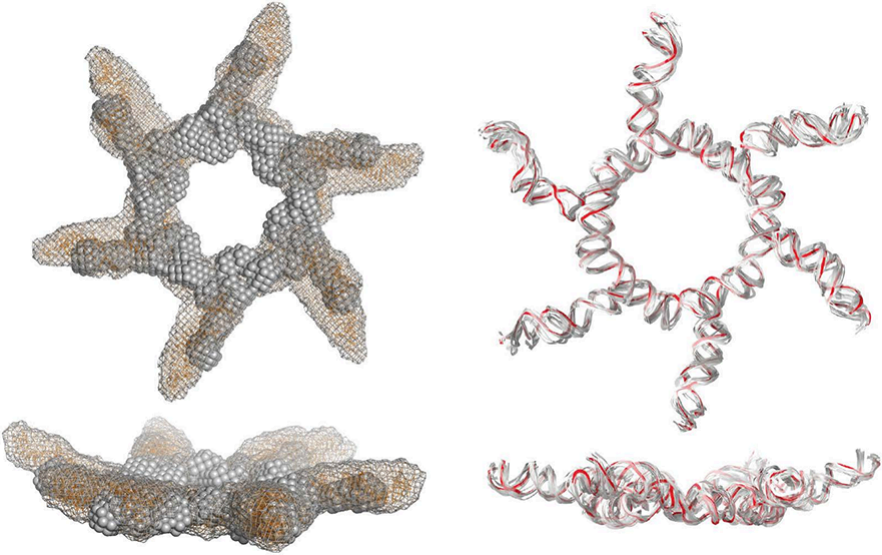

Nucleic acids (RNA and DNA) play crucial roles in all living organisms and find wide utility in clinical settings. The convergence of rationally designed nucleic acid multistranded assemblies with embedded therapeutic properties has led to the development of a platform based on nucleic acid nanoparticles (NANPs). NANPs incorporate various functional moieties to deliver their combinations to diseased cells in a highly controlled manner. Given that the structure and composition of NANPs can also influence their immunorecognition and biological activities, thorough verification of all designs is essential. We introduce an experimental pipeline for small-angle X-ray scattering (SAXS) to gather structural details about the solution-state NANPs assembled from up to 12 RNA strands. To the best of our knowledge, this study represents the largest multistranded RNA nanoassemblies characterized in this manner to date. We show that synchronized implementation of SAXS-driven molecular dynamics simulations reveals the diverse conformational landscape inhabited by these assemblies and provides insights into their immunorecognition. The developed strategy expands the capabilities of therapeutic nucleic acids and emerging nucleic acid nanotechnologies.

## Introduction

In recent years, there has been a surge in developing novel nucleic acid therapies.^1,2^ However, unmodified exogenous RNA and DNA oligos are naturally immunostimulatory and can trigger innate pattern recognition receptors associated with detection of viral and bacterial infections.^3−5^ This presents challenges in achieving efficient delivery of these therapies while minimizing nonspecific toxicity and off-target effects. One way to address these challenges involves incorporating chemically modified nucleobases, as seen in the Pfizer/BioNTech mRNA vaccines.^6,7^ Another promising strategy is the design of self-assembling nucleic acid nanoparticles (NANPs), which can enable coordinated delivery and release of multiple therapeutics while modulating their immunorecogniton.^8−10^ The combination of both approaches is expected to result in functionally sophisticated systems for combinatorial treatments and conditional activation of therapeutic responses upon delivery of a single NANP.^11,12^ With the rapid advancement of these technologies leading to increasingly complex structures, there is an urgent need for new experimental pipelines to offer comprehensive structural characterization of current and prospective designs.

Various therapeutic and functional domains have been integrated into multistranded NANPs, resulting in a repertoire of assemblies with unique geometries and compositions.^13−16^ Despite recent progress in understanding nucleic acids folding, along with increasingly sophisticated computational algorithms, which make *in silico* prediction of NANPs more achievable,^16−21^ addressing the growing demand for experimental validation of their architectural details remains a challenge. Electrophoretic mobility shift assays (EMSA),^22,23^ dynamic light scattering (DLS),^22^ atomic force microscopy (AFM),^24^ and cryogenic electron microscopy (cryo-EM)^25^ are routinely used for the characterization of multistranded NANPs, yet each technique has its own limitations. Specifically, AFM analysis may yield misleading data for large three-dimensional NANPs due to the electrostatic adherence of nucleic acids to the mica surface. Cryo-EM, while simulating a solution environment, lacks a complete understanding of NANPs inherent flexibility. More advanced techniques such as X-ray crystallography^26^ and nuclear magnetic resonance (NMR)^27^ are constrained by static crystalline state and particle size limitations, respectively. While the combination of these techniques is valuable for revealing assembly yields, dispersity, morphology, and sizes, they do not fully capture the solution-state conformational dynamics of NANPs. Although high-resolution scattering techniques such as X-ray crystallography have the capability of providing atomic-level structural information, SAXS techniques are applied to randomly oriented molecules in solution. As such, analyses of these solution scattering data provide structural insights into systems in which inherent flexibility may cause problems for crystallization. Small-angle X-ray scattering (SAXS) may offer additional insights that, when integrated with data from other techniques, can inform better design and structural guidelines for tested NANPs.

SAXS is well poised to further the understanding of the unique dynamic structure–function relationship of NANPs as they exist in solution. In lieu of a crystal, X-ray scattering from nucleic acid samples in solution can provide essential details on the time-averaged ensemble structure as opposed to exact atom positions. Nonetheless, information about the size, shape, compactness, and molecular weight of the scattering molecules are readily available from the scattering data. Consequently, SAXS data offer structural insights into oligomerization states, overall conformation, and flexibility.^28−31^ SAXS has been extensively utilized to confirm the oligomeric state of proteins and organic polymers, and its inclusion has benefited the characterization of highly flexible biomolecular systems.^32−34^ Recent developments in SAXS-driven molecular dynamics (SAXS-MD), which model the solution state structure by integrating experimental SAXS data with force fields, have also offered benefits for studying modeling biomolecular flexibility.^35−37^

**Figure 1:**
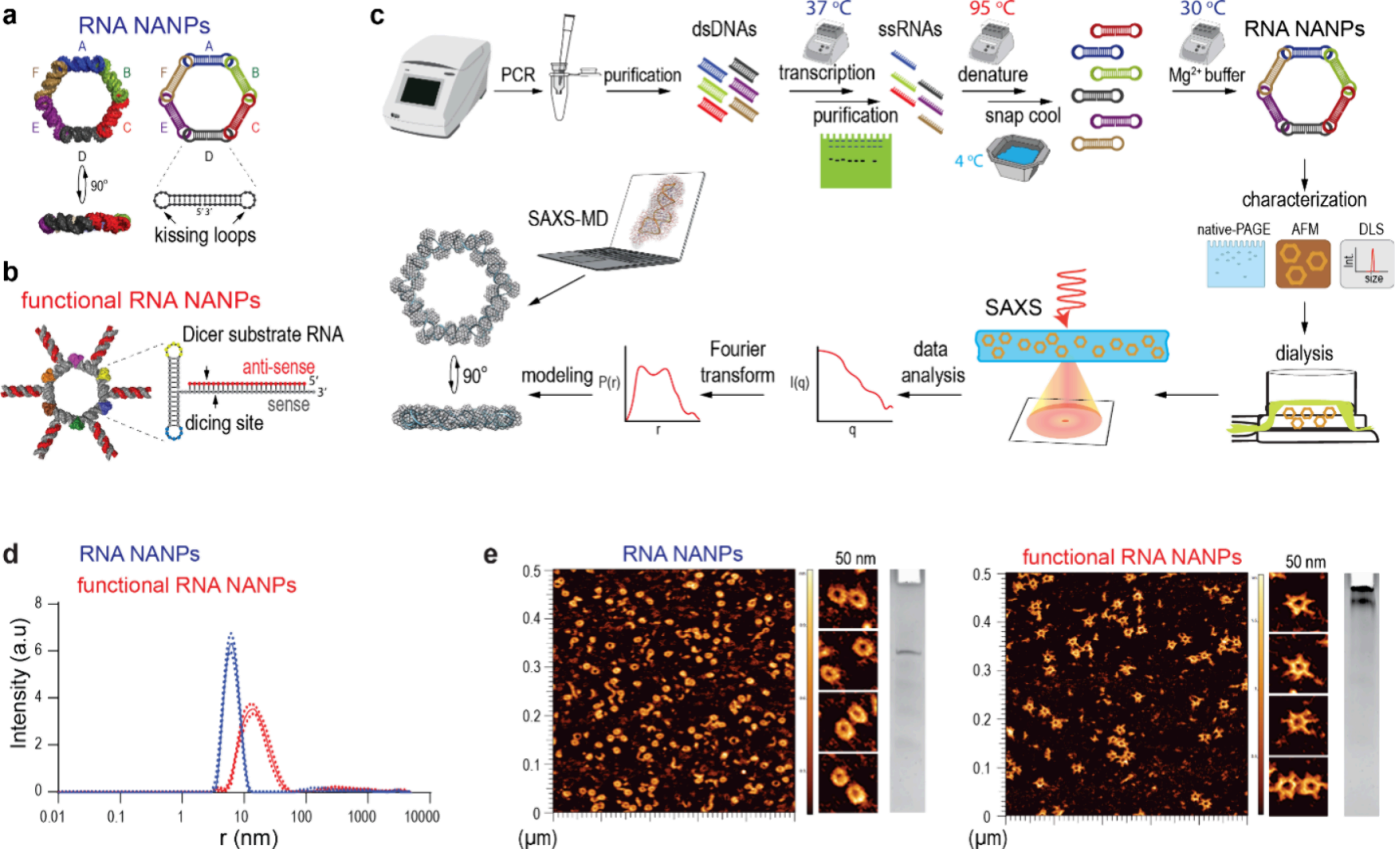
The experimental workflow of the reported work. (a) Nonfunctional six-stranded RNA NANPs and (b) 12-stranded RNA NANPs with six Dicer substrate RNAs. (c) Schematics depicting the synthesis of NANPs, their characterization, and further analysis by SAXS and SAXS-MD. The formation of tested NANPs was confirmed by (d) DLS and using (e) native-PAGE and AFM.

In this study, we introduce an experimental setup for SAXS characterization of a representative RNA NANPs (Figure 1a), illustrated by hexagonal RNA rings.^38^ These nanoassemblies were chosen for their unique combination of secondary and magnesium-dependent tertiary long-range interactions, crucial to the formation of closed, uniformed structures. These computationally designed RNA NANPs assemble via six pairs of colE1-like kissing loops, each forming approximately a 120-degree angle between adjacent helices.^39^ An experimental strategy was implemented to customize each pair of kissing loops located at the sides of the dumbbell-shaped hairpins, enabling programmable six-stranded closed assemblies.^38^ To showcase the versatility and bolster the capabilities of SAXS, we additionally examined RNA NANPs decorated with six Dicer Substrate (DS) RNAs^25,40^ which constitute larger and more complex 12-stranded assemblies (Figure 1b).

The current experimental validation of two tested RNA NANPs was supplemented and directed by SAXS-MD through continuous feedback loop communication. The extensive characterization of RNA NANPs included several conventional approaches such as AFM, CryoEM and SAXS. Importantly, we employ SAXS-Driven molecular dynamics (SAXS-MD) to improve characterization of NANPs in combination with predicted RNA structures and bead models generated from the SAXS data (Figure 1c). Symmetry assumptions were based on the designed structure and supported by AFM and cryo-EM characterizations of this system.^10,25^

## Materials and Methods

### RNA NANP Synthesis and EMSA

All RNA sequences are listed in the SI. Short RNA strands, DNA templates, and DNA primers containing a T7 RNA polymerase promoter region were purchased from IDT. DNA templates for transcription were produced and amplified via PCR using MyTaq Mix (Bioline) and purified using the DNA Clean and Concentrator kit (Zymo Research). *In vitro* runoff transcription was done using T7 RNA polymerase in 80 mM HEPES-KOH buffer (pH 7.5), 50 mM DTT, 2.5 mM spermidine, 5 mM NTPs, and 25 mM MgCl_2_. DNase (Promega) was introduced to stop the transcription. Denaturing polyacrylamide gel electrophoresis (PAGE) with 8 M urea was used to purify the RNAs, which were visualized under a short wavelength UV lamp, excised, and eluted overnight in a solution of 300 mM NaCl, 2 mM EDTA, and 89 mM Tris-borate at pH 8.2 (Sigma-Aldrich). The eluted RNAs were mixed with 2.5 volumes of 200-proof ethanol (VWR), washed twice with 180-proof ethanol, and dried by vacuum centrifugation at 55 °C. The RNAs were resuspended in 17.8 MΩ cm ultrapure water. Corresponding RNA strands were mixed at equimolar concentrations and mixtures were heated to 95 °C for 2 min, then snap-cooled to 4 °C for 2 min. Following cooling, the mixture was incubated at 30 °C for 30 min in assembly buffer (89 mM tris-borate, pH 8.2, 2 mM MgCl_2_, 50 mM KCl). To compare the structures of the nonfunctional RNA monomer without the presence of MgCl_2_, strands were folded in assembly buffer without MgCl_2_ (89 mM tris-borate, pH 8.2, 50 mM KCl). All monomers and assemblies of RNA NANPs were confirmed using a nondenaturing (native) 8% PAGE (37.5:1 acrylamide:bis(acrylamide)), or 1.5% agarose and visualized using ethidium bromide total staining.

### RIG-I Activation

HEK Lucia RIG-I cells, maintained in 1x DMEM (4.5 g/L glucose, 2 mM l-glutamine), 10% heat-inactivated fetal bovine serum, 1% Penicillin-Streptomycin, Normocin (100 μg/mL), Blasticidin (30 μg/mL) and Zeocin (100 μg/mL), were transferred into a sterile 96-well plate (Thermo Fisher Scientific) at 20,000 cells per 100 μL well. Cells were left to incubate overnight (37 °C, 5% CO_2_). The following day, the nonfunctional and functional RNA monomers were mixed with Lipofectamine 2000 (Thermo Fisher Scientific) and incubated at room temperature for 30 min. Media was aspirated from the wells containing the adhered cells and replaced with fresh media containing lipoplexes at a final concentration of monomers of 10 nM. Twenty-four h after transfection, 50 μL of QUANTI-Luc (warmed to 37 °C) were added to 20 μL of cell supernatant in a new black-walled 96-well plate (Thermo Fisher Scientific). The plate was then immediately read for luminescence (100 ms reading time) on a Tecan Spark plate reader. Data was normalized against untreated cells and plotted using GraphPad Prism.

### Atomic Force Microscopy (AFM)

RNA NANPs at different dilutions were placed onto a freshly cleaved mica surface, modified with aminopropyl silatrane, and allowed to dry for 2 min. Unbound RNA was removed by washing the surface twice with 50 μL of deionized water, followed by gently drying with a flow of argon gas. AFM was performed with a MultiMode AFM Nanoscope IV system (Bruker Instruments) using a TESPA-300 probe. Micrographs were recorded with a 320 Hz resonance frequency, 1.5 Hz scanning rate, and a spring constant of 40 N/m in tapping mode. Micrographs were processed with FemtoScan Online (Advanced Technologies Center, Moscow, Russia) as previously reported.^9^

### Dynamic Light Scattering (DLS)

RNA NANPs were 0.2 μm filtered and 40 μL of solution was used for each measurement with plastic micro cuvettes (Malvern Panalytical). Light scattering data was collected using a Malvern Instruments Zeta Sizer Nano with a 633 nm red laser. A total of 5 runs with 15 measurements per run were averaged. The equilibration time was set to 5 min and the scattering signal was collected at a 173° angle and the refractive index was set to 1.45. Data is presented as the Z-average hydrodynamic radius (*R*_h_) ± the standard deviation.

### SAXS

All RNA NANPs were dialyzed against 0.2 μm filtered assembly buffer at 4 °C with 12 h between buffer changes. The third dialysate was used for measurements as the matched buffer. For the monomer experiments, the RNA NANP ‘A’ oligo was used in the nonfunctional case, and “DS-A” with its complementary oligo were used for the DS functional case. The same protocol was used for the preparation of monomer samples using the same buffer solution sans MgCl_2_. SAXS/WAXS data was collected at the Life Science X-ray scattering (LIX) beamline (16-ID) at the National Synchrotron Light Source II at Brookhaven National Laboratory. Data collection employed high throughput static SAXS (HT-SAXS) methods and size exclusion chromatography coupled with SAXS (SEC-SAXS). Briefly, 60 μL of sample was placed into PCR tubes along with matched buffer and then into a custom LIX holder.^41^ Samples were placed in the experimental end station, aspirated, and flowed through a flow cell placed in the path of the X-ray beam. SAXS/WAXS were collected simultaneously with an exposure time of 1 s and 5 frames (repeats). Data were averaged, scaled, and merged as previously described.^41^ The data collected from the functional RNA NANPs was previously published using a different analysis methodology as part of a separate study.^10^ For the static experiments, flow rate was 9 μL/sec. Similarly, SAXS/WAXS data were collected on nonmonodisperse samples using SEC-SAXS. Separation was performed using an Agilent 1260 Infinity II Bio Inert HPLC system. 50–60 μL of sample was injected using the Agilent multisampler, in-line with a Superdex 200 Increase 5/150 GL column (Cytiva) with a flow rate of 0.35 mL/min (5.8 μL/sec). The column was equilibrated in several column volumes of sample buffer. X-ray Energy was 15.16 kEV with a wavelength of 0.819 Å. SAXS/WAXS detectors were Pilatus 1 M and Pilatus 900 K respectively. Data analysis and visualization was performed at the LIX beamline using custom py4xs and lixtools software, freely available on Github.^41−43^ Matched buffer data were recorded using the final dialysate and subtracted from the sample measurements and normalized to the water peak at 2.0 Å^–1^ to yield the scattering profile of each particle. PRIMUS software was used to yield the root-mean-square distance of each atom to the center of mass, radius of gyration (*R*_g_), through a Guinier approximation using the low-q data.^44^ The pairwise probability distance distribution, P(r), and *R*_g_ were calculated and *d*_max_ was estimated using the GNOM software (ATSAS 3.0.1).^45^ Bead models of the monomer units were generated using DAMMIF to generate 20 models, superimpose, average them, and the resultant volume minimized model from DAMFILT is shown.^46^ Briefly, DAMMIF generated bead models based on criteria defined from the GNOM output such as the distance distribution function, which provides the maximal particle size and box that the bead model must fit. Beads were randomly assigned to either solvent or solute (RNA in this case) and a scattering profile was generated. Beads were then randomly changed in phase and a new scattering profile was generated and assigned a probability. This was iterated until convergence was reached by minimizing a χ^2^ fit to data and restraining parameters such as chain connectivity between beads that were assigned to solute, *R*_g_ etc. The scattering of the models was fit to the experimental scattering profile using CRYSOL.^47^ The secondary structure of the monomeric subunits was predicted using NUPACK.^21^ The predicted secondary structure was analyzed with RNA Composer to predict the three-dimensional structure of each subunit.^48^ To model the assembled NANPs, the DAMFILT models of each monomer were used for rigid body modeling using SASREF with 6-fold rotational symmetry as the only structural constraint.^49^

### SAXS-MD

SAXS-MD aims at modeling the solution state structure of the biomolecule by mimicking a SAXS experimental set up *in silico*. It is a three-part process which requires a free MD simulation (unconstrained MD) of RNA NANPs in solvent for determining the force field based conformational space of the RNA molecules, a solvent explicit simulation for buffer subtraction, and an experimental data-constrained MD simulation of RNA NANPs in a solvent which together with force fields drives the simulation toward a true solution state structure.^35−37^ All three simulations were performed using the Amber force fields^50,51^ (AMBER03) with TIP3P water model^52^ using the modified version of GROMACS known as GROMACS-SWAXS (https://gitlab.com/cbjh/gromacs-swaxs).^35^ The free-MD simulation was performed as previously described.^53^ Briefly, the RNA in solvent system was neutralized by adding 2 mM MgCl_2_ and 50 mM KCl for RNA NANPs and functional RNA NANPs, and sans MgCl_2_ for both nonfunctional RNA monomer and functional RNA monomer followed by energy minimization and 20 to 100 ns production MD at constant temperature and pressure (300 K and 1 atm respectively). Two explicit solvent simulations were performed both with and without MgCl_2_ to match the solvent conditions for RNA NANPs and RNA monomers, respectively. The SAXS-MD simulation converted the SAXS experimental data into potentials to drive the conformation space toward a true solution state structure. The predicted RNA models from RNAComposer^48,54^ were used as the starting structure for SAXS-MD where the SAXS-driven forces were turned on gradually in the simulation defined by waxs-t-target (3.5 to 5 ns for different RNA molecules based on size). The force constant was set to 1 for Bayesian inference and the memory time (waxs-tau) was set to 250 ps. On the fly curves, which depict the calculated SAXS intensities in the last 250 ps of the simulation, were assessed for their fits against the experimental data. Moreover, to calculate average SAXS intensities for the ensembles obtained from the complete simulation trajectory, the RERUN module of the GROMACS-SWAXS package was employed. This module calculated the SAXS intensities at all time steps in the simulation and generates an average SAXS intensity profile as well as calculated the Guinier *R*_g_ from the average SAXS profile. The standard parameters for SAXS-MD, such as target time for gradually including SAXS potentials (waxs-t-target), nature of potentials (log or linear), nature of coupling weights, coupling time constant (waxs-tau), number of q points (nq), solvent uncertainty (waxs-solv-uncertainty), *etc*., were obtained as described in the SAXS-MD tutorial book chapter.^55^ Since the sizes of each of the four samples were different, some of these parameters were further adjusted based on the total size of the simulation and are listed in Table S1. The final output from the SAXS-MD simulation consisted of a trajectory of the simulation, average SAXS intensities from the RERUN module as well as spectra of SAXS intensities at every time step in the simulation which can be analyzed along with the experimental data to identify at which time step the simulation converges (i.e., the calculated SAXS intensities are the closest to the experimental intensities). Based on the analysis of spectra of calculated SAXS intensities against the experimental data, multiple models were extracted from the SAXS-MD trajectory after the SAXS potentials were included in the simulation (waxs-t-target), and WAXSIS server (https://waxsis.uni-saarland.de/) was used to calculate the model to data fit plots for the best fitting individual models.^56^ Based on the χ^2^ value for each of the individual models, the top 10 best fitting models were selected for visualizing the RNA NANP structures along with the bead models derived from the SAXS data. The SAXS-MD model with the lowest χ^2^ value (as obtained from the WAXSIS server) as compared to the predicted RNA structure was designated as the best fitting model. The models were visualized using PyMol^57^ and ChimeraX.^58^

The stability of the simulations was analyzed by calculating Root Mean Square Deviation (RMSD) and *R*_g_ over time from the trajectory files. For determining the backbone flexibility (global changes), Root Mean Square Fluctuations (RMSF) per nucleotide base were calculated averaged over time from the trajectory files. The residues which showed RMSF values above the 75th-percentile threshold were considered to have the maximum impact during the simulation and if 2 or more of such consecutive residues were observed, they were grouped together to identify regions of importance in the overall structure model. The local structural changes were analyzed using the ribose sugar pucker parameters, specifically the phase angle and amplitude. The phase angle, which ranges from 0° to 360°, describes the specific conformation of the ribose ring, with values around 18° indicating C3′-endo (characteristic of A-form RNA) and values around 162° indicating C2′-endo conformations. The amplitude represents the degree of ring puckering, with larger values indicating more pronounced nonplanar conformations. These parameters were calculated using the five endocyclic torsion angles (ν0-ν4) of the ribose ring for each residue throughout the trajectory. The distributions of both phase angles and amplitudes were visualized as density plots, where the *x*-axis represents either the phase angle in degrees or the pucker amplitude, and the *y*-axis shows the probability density of each conformation throughout the simulation. Residues showing distinct multimodal distributions or peak shifts greater than 2σ (two standard deviations) from the mean values in either phase angle (from C3′-endo ∼18°) or amplitude distributions were identified as having significant conformational changes. Particularly noteworthy were residues exhibiting either complete conformational switches (peaks around both C3′-endo and C2′-endo regions) or substantial deviations in pucker amplitude indicating altered ring geometries. This combined analysis of global (RMSF) and local (sugar pucker) dynamics provided insights into both backbone flexibility and specific conformational changes in the RNA structure. The trajectories were analyzed using in-house codes developed with MdTraj,^59^ a python library for analyzing molecular dynamics libraries. The electrostatic charge distribution on the RNA structures was calculated using the Adaptive Poisson–Boltzmann Solver (APBS) plugin in Pymol.^60^

## Results and Discussion

### Initial Characterization of Tested RNA NANPs

Based solely on the designed structure, calculations estimate the radius of the RNA NANPs to be around 70 Å, with a maximum linear dimension (*d*_max_) close to 150 Å.^38^ Functionalization with DS RNAs was anticipated to increase the *d*_max_ by approximately 130–140 Å, assuming all helices adopt an A-form and DS RNAs are perpendicular to the sides of the RNA NANP. The hydrodynamic radius (*R*_h_) obtained from DLS reflects the radius of a hard sphere diffusing at a similar rate as the analyte. However, for RNA NANP shapes significantly deviating from spherical shapes, *R*_h_ becomes a less reliable indicator of true size. In our case, the *R*_h_ for tested NANPs is determined to be 53 ± 2 Å, while functional RNA NANPs exhibit a *R*_h_ of 101 ± 3 Å (Table 1 and Figure 1d). Interestingly, the size of functional NANPs obtained from DLS differs from the dimensions observed in AFM micrographs, where *d*_max_ was slightly over 300 Å. Nevertheless, the DLS data confirms that all RNA NANPs assembled into monodisperse populations in solution, as evidenced by their low polydispersity indices. AFM results further validate the assembly of each NANP and justify the imposition of 6-fold rotational symmetry on the system for three-dimensional modeling of SAXS data. Slight structural variations between individual RNA NANPs observed in AFM images are attributed to their fixation onto the mica substrate or interactions with the probe^28^ (Figure 1e).

**Table 1:** Dimensions of the RNA NANPs, Functional RNA NANPs, and Monomers Measured by DLS and SAXS

		Nonfunctional RNA monomers	Functional RNA monomers	RNA NANPs	Functional RNA NANPs
DLS	Z-Average *R*_h_ (Å)	–	–	53 ± 2	101 ± 3
	Polydispersity Index	–	–	0.22 ± 0.03	0.23 ± 0.02
SAXS	Guinier *R*_g_ (Å)	21.2 ± 0.2	38.8 ± 0.6	60.5 ± 0.9	82.2 ± 0.7
	P(r) *R*_g_ (Å)	21.5 ± 0.1	39.2 ± 0.4	57.5 ± 0.3	83.3 ± 0.3
	*d*_max_ (Å)	68	130	166	284

### SAXS Analysis of Nonfunctional RNA Monomers

**Figure 2:**
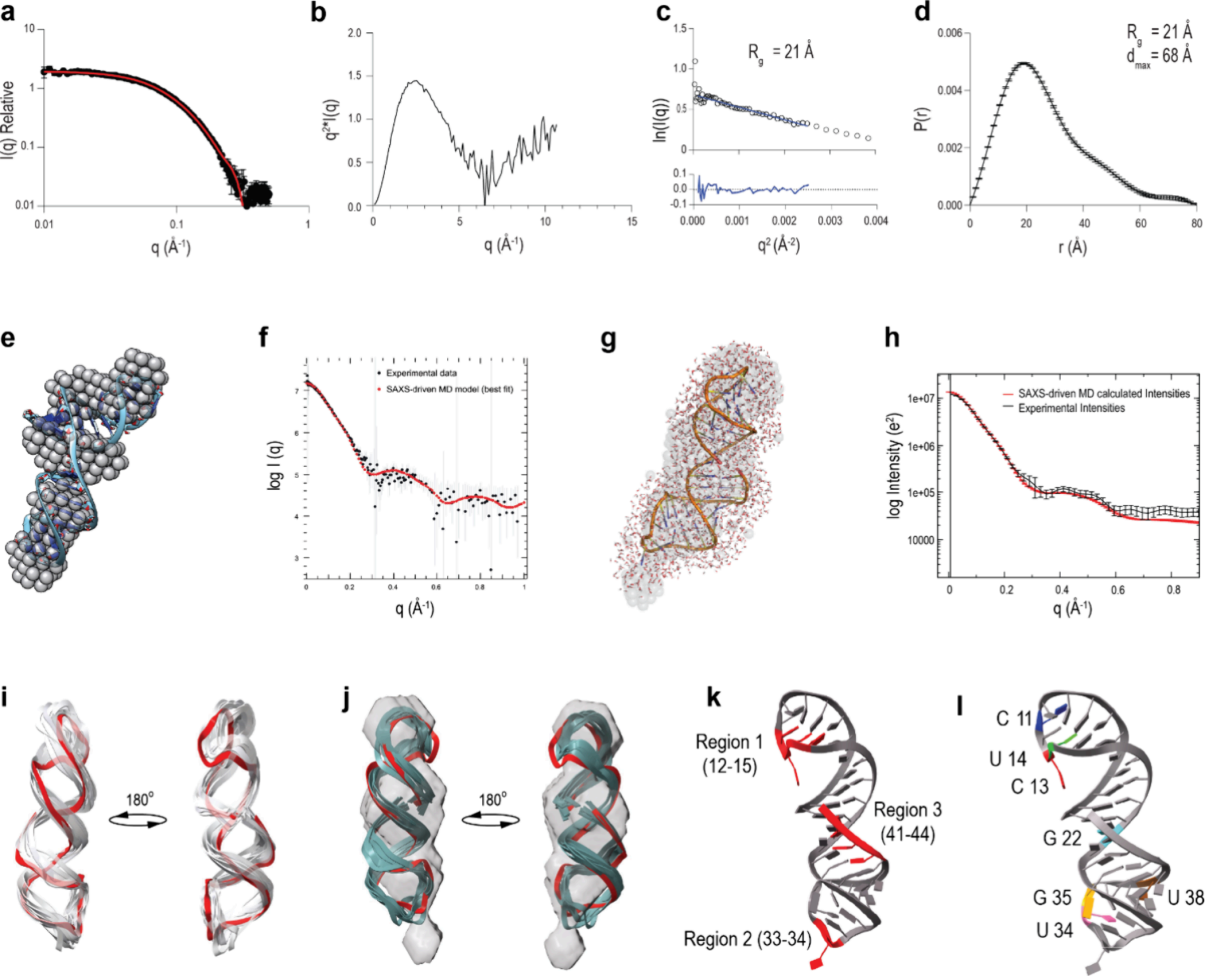
SAXS analysis of the nonfunctional RNA monomers. (a) SAXS profile is shown as black dots with the calculated scattering of the bead model (e) shown as a solid red line (0.025–0.375 Å^–1^). (b) The Kratky plot of the scattering profile. (c) The Guinier fit of the data with the linear fit appearing as a solid blue line. The residuals of the fit are plotted directly underneath the Guinier plot. (d) The pairwise distance distribution of the SAXS data, calculated over 0.025–0.375 Å^–1^. (f) SAXS-MD best fit model obtained from 30 ns simulation fit against the SAXS data using WAXSIS server. (g) The bead model, which is an average of 20 models, with the atomic model generated by SAXS-MD superimposed using SUPCOMB. (h) Average SAXS intensities calculated using the RERUN module of GROMACS-SWAXS (10 ns to 30 ns SAXS-MD trajectory). (i) Top 10 representative models extracted from last 5 ns of 30 ns simulation (gray) with best fit structure in red. (j) Top 10 representative models (cyan) superimposed onto bead model (gray), converted to surface representation in ChimeraX, with best fitting model in red. (k) SAXS-MD model, with three key dynamic regions highlighted: Region 1 (residues 12–15), Region 2 (residues 32–33), and Region 3 (residues 41–44). (l) SAXS-MD model highlighting residues showing distinct sugar pucker conformational deviations, with residues 11(C), 13(C), 22(G), 35(G), 38(U), 34(U), and 14(U) displayed in distinct colors.

To resolve the complete structure of the chosen RNA NANPs, we start with the characterization of individual monomer units. Despite harboring distinct sequences of nucleobases, these monomers exhibit a conserved global structure resembling a dumbbell-like structure with a nick in the middle. Given that the presence of Mg^2+^ ions is required for the formation of kissing loop complexes, all SAXS measurements of the monomers are conducted in a Mg-free buffer. The nonfunctional RNA monomers exhibited signs of polydispersity during initial SAXS/WAXS batch measurements, which results in low quality SAXS data. Therefore, we employed SEC-SAXS to separate out the species and used the monodisperse peak for downstream analysis (SI Figure S1). Each RNA monomer is engineered to self-fold, creating 15 base pair helices with two seven-nucleotide single-stranded loops on each side. The *d*_max_ derived from the pairwise distance distribution closely aligns with the predicted size.^38^ The peak in the P(r) curve at 21 Å is close to what is expected for an RNA helix (Figure 2d).^61^ The reduced χ^2^ of the bead model of the SAXS profile against the experimental data is 0.76, utilizing a q range from 0.011 to 0.25 Å^–1^.^62^ The predicted structure of the monomer, as generated by RNA composer, exhibits a bent conformation, suggesting a diverse array of potential conformational states in solution for the two hairpins. This versatility is anticipated due to the presence of a nick in the middle of the double-stranded region in the dumbbell-like structure. The flexibility of the monomer is supported by the SAXS data, as evidenced by the increase in the low q region of the Guinier range (Figure 2c) and the consistent trend of the Kratky plot beyond the maximum q^2^*I(q) value (Figure 2b). The ensemble of models obtained from SAXS-MD starting with the predicted structure fits well against the SAXS data as shown through the average SAXS intensity plot in Figure 2h.

Analysis of the SAXS-MD trajectory revealed key dynamic regions in the nonfunctional RNA monomers. The RMSD and *R*_g_ plots exhibited several local minima during the simulation trajectory, but notably achieved stable values toward the end of the 30 ns simulation, indicating convergence of the simulation (SI Figure S2A). Analysis of RMSF identified three key dynamic regions, Region 1 (residues 12–15), Region 2 (residues 32–33), and Region 3 (residues 41–44), all exhibiting RMSF values above the 75th-percentile threshold (∼0.825 nm) (SI Figure S2B). The RMSF analysis identified regions of enhanced flexibility, particularly in the kissing loop regions, as highlighted in Figure 2k. Furthermore, local conformational analysis through sugar pucker phase angles showed that while most residues maintained typical A-form RNA C3′-endo conformation (peaks around −50° to 0°) (SI Figure S2C), several residues, particularly residues 11(C), 13(C), 22(G), and 35(G), displayed distinct conformational preferences (Figure 2l). 22(G), positioned at a crucial junction in the structure, exhibited significant conformational flexibility along with residues 11(C) and 13(C), while 35(G) showed a notable shift toward C2′-endo conformation. These deviations from the canonical structure, particularly at 22(G) which could influence the overall topology of the RNA, suggest important local structural variations that may impact the molecule’s functional dynamics.

The best fitting SAXS-MD model (based on the χ^2^ value) fits well against the SAXS data (Figures 2f-g) as compared to the predicted RNA model (SI Figure S3A). The χ^2^ value for the predicted structure obtained from WAXSIS server was less than 1 (χ^2^ = 0.92) whereas for the SAXS-MD model was equal to 1. The bend conformation observed from the predicted structure is not observed after SAXS-MD (SI Figure S3B), alluding that the nick in the middle of the RNA does not play a significant role in the ensemble of models obtained from SAXS-MD. The Guinier *R*_g_ calculated through both on the fly curve (SI Figure S3D) and average SAXS curve from SAXS-MD (Figure 2h), is 20 Å, which agrees with the experimental data. The average SAXS intensities obtained from the RERUN module of GROMACS-SWAXS encapsulate all the conformations (ensemble) observed through SAXS-MD and therefore fit better with the experimental SAXS data as compared to individual models extracted from the SAXS-MD trajectories as shown in Figure 2h. For identifying the regions specifically exhibiting flexibility, the top 10 best fitting structures extracted from a 20 ns SAXS-MD trajectory were superimposed (gray with best fitting model shown in red); as shown in Figure 2i.

The best fitting model derived from SAXS-MD is superimposed with the bead model obtained from DAMMIF using SUPCOMB. The resulting Normalized Spatial Discrepancy (NSD) value of 1.3, exceeding 1, suggests significant disparity between the two models. While the visually apparent fit of the best-fit SAXS-MD model onto the bead model appears satisfactory, as depicted in Figure 2g, it is noteworthy that the bead model only pertains to 0.25q, while the SAXS-MD simulation encompasses higher q data. Hence, the distinctions observed may be attributed to the finer details captured by the SAXS-MD. The overall structure analysis of the SAXS-MD trajectories and their superimposition with the bead model depict that the kissing loop regions contribute to toward the flexibility of the molecule, whereas the region closest to the nick is more stable (Figure 2j-l).

Additionally, to validate that the SAXS data is indeed driving this conformational change in the molecule and not the forcefields alone, we perform an extended unconstrained free-MD simulation of 100 ns. The analysis of the free-MD trajectory shows that the bend region (region close to residue 22(G)) is not completely resolved (SI Figure S3C(ii)). A comparison of model to data fit from the last frame of free-MD simulation (χ^2^ = 3.1) with SAXS-MD best fitting model (SI Figure S3C(i)) shows a better convergence against the SAXS data from the SAXS-MD simulation (χ^2^ = 1). This comparison not only confirms the applicability of such data constrained MD simulations for biomolecules that exhibit flexibility, but also reduces the overall computation time and cost (100 ns Free-MD versus 30 ns SAXS-MD) to obtain a more realistic solution structure.

### SAXS Analysis of Functional RNA Monomers

**Figure 3:**
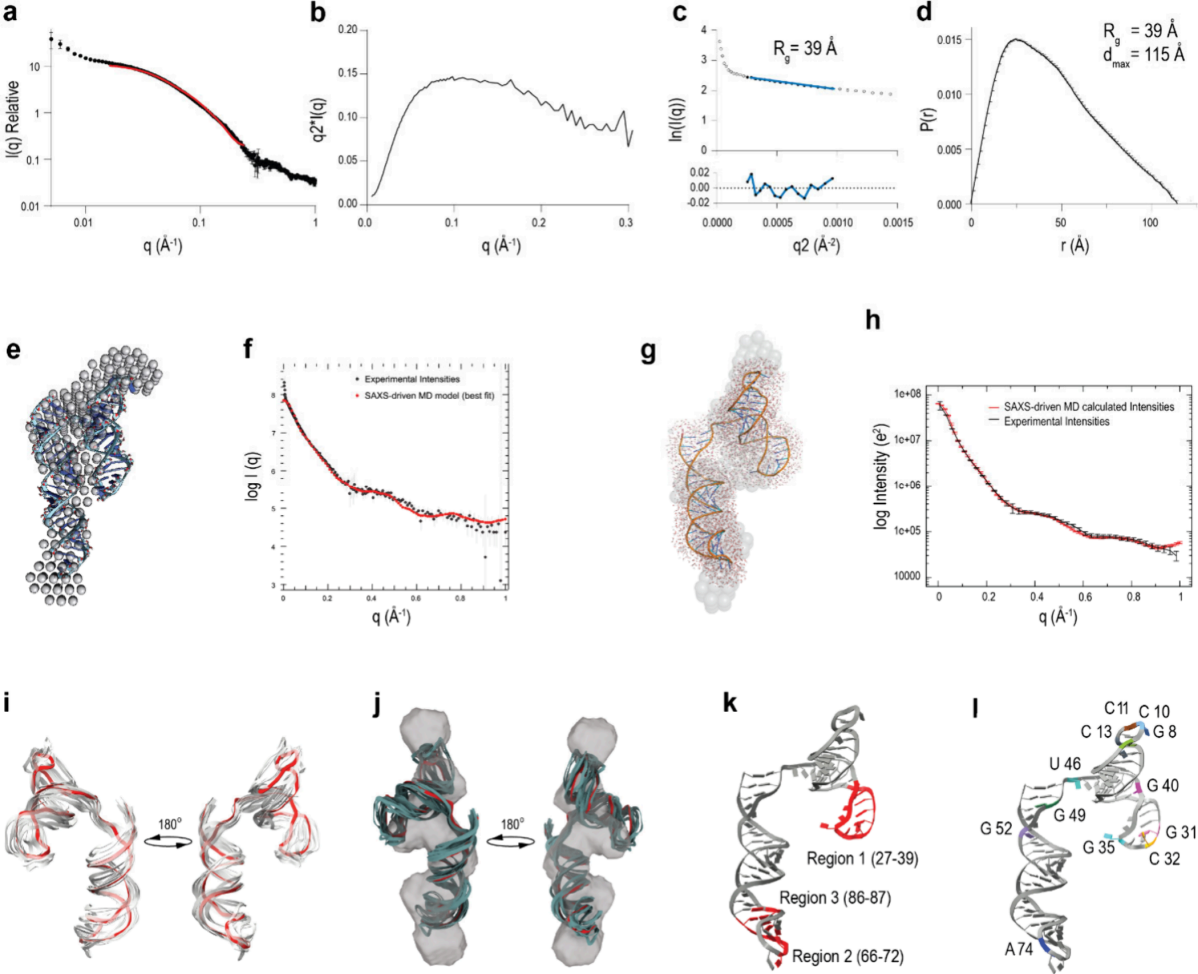
SAXS analysis of the functional RNA monomers. (a) The experimental SAXS profile of the DS RNA monomer is shown as black dots with the calculated scattering of the bead model (e) shown as a solid red line (0.016–0.25 Å^–1^). (b) The Kratky plot of the scattering profile. (c) The Guinier fit of the data with the linear fit appearing as a solid blue line. The residuals of the fit are plotted directly underneath the Guinier plot. (d) The pairwise distance distribution of the SAXS data, calculated over 0.016–0.25 Å^–1^. (f) SAXS-MD best fit model obtained from 15 ns simulation fit against the SAXS data using WAXSIS server. (g) The bead model, which is an average of 20 models, with the atomic model generated by SAXS-MD superimposed using SUPCOMB. (h) Average SAXS intensities calculated using the RERUN module of GROMACS-SWAXS (5 to 15 ns SAXS-MD trajectory). (i) Top 10 representative models extracted from last 5 ns of 15 ns simulation (gray) with best fit structure in red. (j) Top 10 representative models (cyan) superimposed onto bead model (gray), converted to surface representation in ChimeraX, with best fitting model in red. (k) SAXS-MD model, with three key dynamic regions highlighted: Region 1 (residues 27–39), Region 2 (residues 66–72), and Region 3 (residues 86–87). (l) SAXS-MD model highlighting residues showing distinct sugar pucker conformational deviations, with residues displayed in distinct colors as per the legend in SI Figure S5C.

The functional RNA monomer shares the same sequence for forming the dumbbell structure as the nonfunctional RNA monomer. However, it is elongated at the 3′ end by a 29-nucleotide sequence that is complementary bound to a 25-nucleotide long RNA oligonucleotide, resulting in the formation of a DS RNA with a 2-nucleotide 3′-side overhang and a 2-nucleotide long linker. As anticipated, the resulting structure is significantly larger than the nonfunctional RNA monomer, with a *d*_max_ 40 Å longer. Additionally, its *R*_g_ nearly doubles compared to the DS RNA monomer unit. From the Kratky plot (Figure 3b), it is evident that the RNA NANP remains highly flexible, which is expected due to the presence of two unpaired nucleotides linking the DS RNA to the dumbbell. DAMMIF was run using a q range of 0.015 to 0.2 Å ^–1^ with a χ^2^ of 0.944. Including higher q data for the bead models results in poor fits of the model to SAXS profiles as is demonstrated by high χ^2^ values. The ensemble models obtained from SAXS-MD (Figures 3h) fit well against the SAXS data.

The functional RNA monomer, as predicted using RNAComposer, is depicted as to be compact with the DS sequence aligning alongside the dumbbell forming sequence of RNA monomer, shown in SI Figure S4B(i). The calculated *R*_g_ for the predicted structure is 26 Å, indicating that the predicted structure is more compact than anticipated in the solution state. Contrasting with the predicted RNA model, the SAXS-MD model illustrates flexibility in the linker region, (SI Figure S4B(ii)) with a predicted *R*_g_ of 34.4 Å thus depicting an extended form. The top 10 best fitting models extracted from the SAXS-MD trajectory and the best model fits better against the SAXS with a χ^2^ value of 2.2 (Figure 3f-g) as compared to that against the predicted RNA structure with a χ^2^ value of 6.7 (SI Figure S4A).

Analysis of the SAXS-MD trajectory revealed interesting dynamic behavior throughout the simulation. The RMSD and *R*_g_ plots exhibited continuous increase even after the waxs-t-target time point (time until which SAXS data was incorporated in the simulation), indicating that the structure did not achieve complete stability during the 15 ns simulation (SI Figure S5A). This continuous increase in both parameters, particularly evident in the latter part of the trajectory, indicates that the functional RNA monomer samples a broader conformational space and may require enhanced sampling techniques such as multiple replica exchange molecular dynamics or longer simulation times to achieve a stable trajectory.

From the current simulation trajectory, RMSF analysis identified three key dynamic regions highlighted in red in Figure 3k *i.e*. Region 1 (residues 27–39), Region 2 (residues 65–72), and Region 3 (residues 86–87), all exhibiting RMSF values above the 75th-percentile threshold (SI Figure S5B). Local conformational analysis through sugar pucker phase angles revealed several residues with distinct conformational preferences (SI Figure S5C). Notably, residues C10, C11, C13, and G8 in the first stem region, along with G40, U46 and G49 in the central region, showed significant deviations from typical A-form RNA conformations as highlighted in Figure 3l. The sugar pucker amplitude distributions further supported these findings, with these residues displaying broader distributions, indicating greater conformational flexibility.

The Guinier *R*_g_ calculated through both on the fly curve (SI Figure S4C) and average SAXS curve from SAXS-MD (Figure 3h) respectively, is approximately 35 Å, which represents the average *R*_g_ observed during the entire simulation. This value deviates from the experimentally calculated *R*_g_ of approximately 39 Å from Guinier and P(r) analysis. Although we observe the extension of the functional RNA monomer during the simulation where the *R*_g_ increases from 26 Å (of the predicted model) to 34.4 Å (SAXS-MD best model), there are a few factors for not achieving the desired *R*_g_ of 39 Å from the simulation. First, these experimental data are not linear in the Guinier region and shifted to higher q and are therefore not representative of the true *R*_g_ value. Given the flexibility, Guinier analysis is not the best tool to calculate *R*_g_ in this case, since multiple shapes explain these data. Second, the SAXS data undergoes smoothing before being utilized in the simulation, resulting in the exclusion of several points at low q, which represent the flexibility of the DS monomer (supported by the Kratky analysis in Figure 3b). Additionally, the size of the envelope defined around the predicted model may need to be enlarged to accommodate the extension by up to 40 Å, necessitating a longer simulation time and cost.

Nonetheless, the trajectory observed during the SAXS-MD provides insight into the flexibility imparted by different regions, which is adequate for approximating the solution state of the molecule. The top 10 best-fitting structures extracted from a 15 ns SAXS-MD trajectory are superimposed (gray, with the top model shown in red), as depicted in Figure 3i. These structures illustrate that the entire molecule exhibits flexibility, including the kissing loop region of the RNA monomer and the linker region of the DS RNA sequence. To visually verify the alignment, the best fitting model obtained from SAXS-MD is aligned with the bead model, as depicted in Figure 3g, using SUPCOMB, as previously described. NSD value of 2.59 suggests systematic differences between the two models. This is expected, as no single structure can fit well against the SAXS data for such a flexible molecule; however, the top 10 structures superimposed onto the bead model, shown in surface representation Figure 3j, reveal the overall flexibility in the molecule and the models fill in the volumetric space depicting an overall similar shape.

### SAXS Analysis of the Nonfunctional RNA NANPs

**Figure 4:**
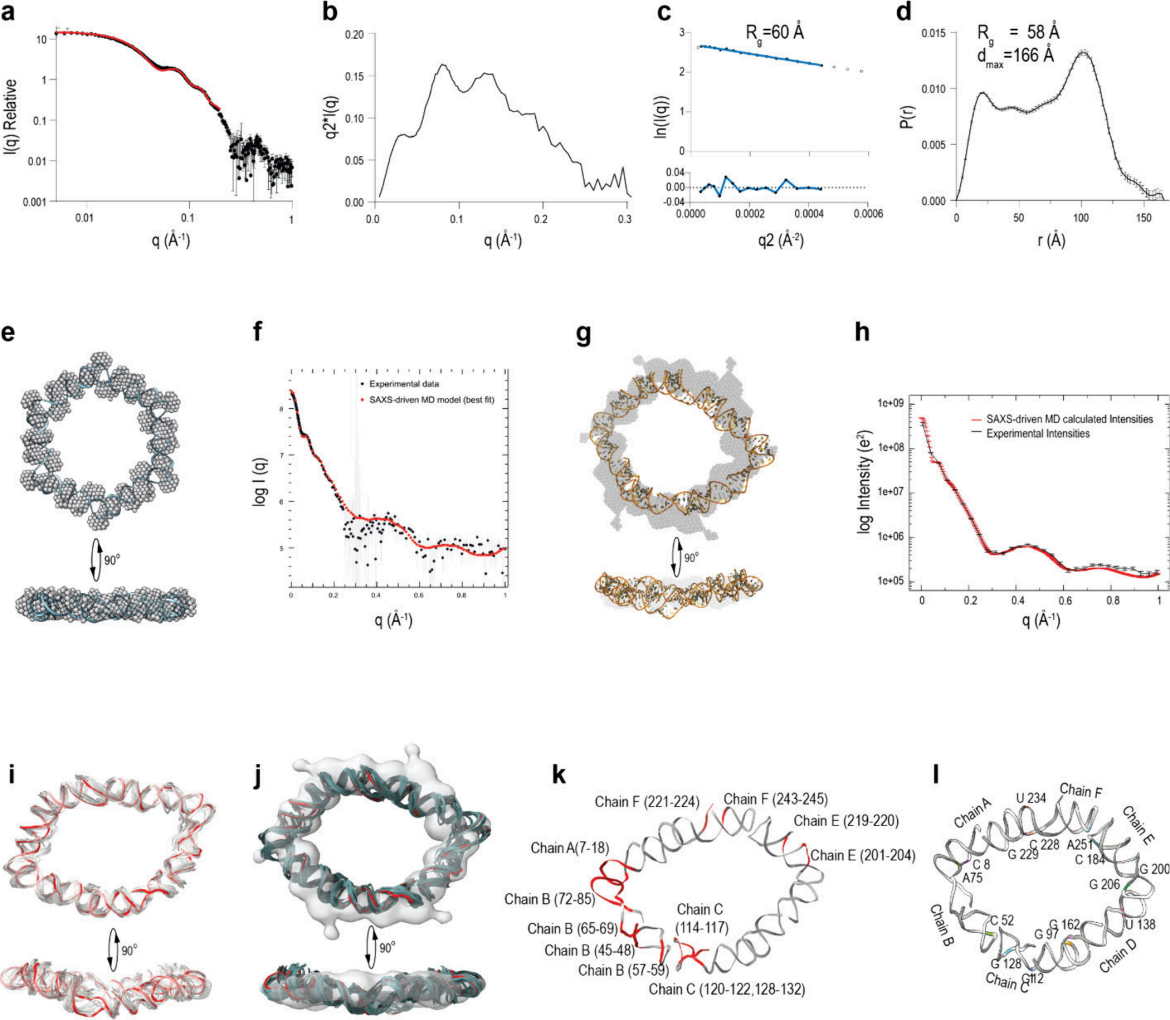
SAXS analysis of the nonfunctional RNA NANPs. (a) The experimental SAXS profile of RNA NANPs shown as black dots with the calculated scattering of the bead model (e) shown as a solid red line. (b) The Kratky plot of the scattering profile. (c) The Guinier fit of the data with the linear fit appearing as a solid blue line. The residuals of the fit are plotted directly underneath the Guinier plot. (d) The pairwise distance distribution of the SAXS data, calculated over 0.005–0.2 Å^–1^ is shown. (f) SAXS-MD best fit model obtained from 12 ns simulation fit against the SAXS data using WAXSIS server. (g) The bead model generated using rigid body modeling (SASREF) of the monomer to fit the experimental data with P6 symmetry is shown with the anticipated three-dimensional structure superimposed. (h) Average SAXS intensities calculated using the RERUN module of GROMACS-SWAXS (5 to 12 ns SAXS-MD trajectory). (i) Top 10 representative models extracted from last 5 ns of 12 ns simulation (gray) with best fit structure in red. (j) Top 10 representative models (cyan) superimposed onto bead model (gray), converted to surface representation in ChimeraX, with best fitting model in red. (k) SAXS-MD model, with key dynamic regions highlighted as per the legend in SI Figure S7B. (l) SAXS-MD model highlighting residues showing distinct sugar pucker conformational deviations, with residues displayed in distinct colors as per the legend in SI Figure S7C.

The RNA NANPs shows an *R*_g_ which is slightly larger than the *R*_h_, determined through DLS measurements, giving an *R*_g_/*R*_h_ of 1.08–1.14 (Table 1). This ratio being greater than 1 is expected for particles that deviate significantly from spherical, which would have a *R*_g_/*R*_h_ close to 0.78.^63^ While the constituent monomers of the RNA NANPs are shown to be flexible through their Kratky plots (Figures 2 and 3b), the fully assembled RNA NANPs do not seem to have the same degree of flexibility despite each monomer having the nick in the middle of its double-stranded region (Figure 4b). The RNA NANPs appear to be slightly larger than the predicted *d*_max_ of 150 Å.^38^ This may be due to a combination of the RNA NANPs having a “breathing” mode of movement in solution to adopt a slightly elongated conformation, and the attraction of a large ion and hydration layer around the highly charged RNA backbone.^64,65^

SAXS-MD performed on the RNA NANPs supports this observation of a breathing motion which leads to a slightly elongated conformation with changes at the kissing loop junctions. The best fit model obtained from SAXS-MD trajectory analysis (Figure 4f-g) shows a slightly better fit (χ^2^ value of 2.1) than the initial predicted model (χ^2^ value of 2.5) (SI Figure S6A). The single model represented in Figure 4f-g does vary slightly in the high q region from the experimental SAXS data, depicting that no single structure can truly represent the solution state of the RNA NANP. It is therefore essential to consider the average SAXS intensities calculated from the ensemble models represented through the SAXS-MD trajectories, and how well they fit against the experimental SAXS data. The Guinier Rg calculated from both on the fly curve (SI Figure S6C) as well as average SAXS curve (Figure 4h) is approximately 60 Å, which coincides with the experimental *R*_g_. The SAXS-MD model (SI Figure S6B(ii)) depicts an elongated conformation with kissing loop junctions stretched at opposite sides (marked with black arrows) as compared to the predicted model (SI Figure S6B(i)).

The assembled RNA nanoring MD trajectory showed mixed stability characteristics during the simulation. While the RMSD values initially increased and then stabilized after 8 ns, suggesting overall simulation convergence, the *R*_g_ exhibited more pronounced fluctuations, ranging between 57.5 and 60.0 Å throughout the 12 ns trajectory (SI Figure S7A). This behavior indicates that while the individual structural elements maintain their integrity, the complete ring structure undergoes breathing motions that affect its global dimensions. RMSF analysis revealed several dynamic regions distributed around the nanoring structure, with distinct patterns in each of the six chains (A-F) (SI Figure S7B). The most prominent flexible regions are highlighted in red in Figure 4k, such as residues 7–18 in Chain A, 65–69 in Chain B, 114–117 in Chain C, and corresponding regions in Chains D-F, all exhibiting RMSF values above the 75th-percentile threshold. These regions primarily correspond to the kissing loop interfaces between adjacent chains also suggesting their role in the ring’s breathing motion.

Local conformational analysis through sugar pucker phase angles revealed specific residues with distinct conformational preferences (Figure 4l). Notably, residues at the chain junctions: C8 (Chain A); A75 and C 52 (Chain B); G128 and G97 (Chain C); G162 (Chain D); G206 and C184 (Chain E) and A251, G229 and U234 (Chain F) showed significant populations of noncanonical conformations, as evidenced by their phase angle distributions. The sugar pucker amplitude distributions further supported these observations, with these key residues showing broader distribution patterns indicating enhanced conformational flexibility as shown in SI Figure S7C.

The distribution of dynamic regions and the specific residues showing conformational variations suggests a coordinated motion where the nanoring structure “breathes” through synchronized movements at the chain interfaces. This flexibility appears essential for maintaining the overall ring architecture while allowing for necessary structural adaptations. This is further supported by our bead modeling approach using SASREF with enforced P6 symmetry, a constraint substantiated by the design of this NANP which guarantees complete assembly only when all six constituent strands are present. The symmetry constraint is also validated by AFM micrographs depicting hexagonal RNA NANPs (Figure 1e).

The top 10 best models extracted from the SAXS-MD trajectory depict stretching at the kissing loop junctions of chains A:B and D:E through this Bayesian fit modeling (Figure 4i). Notably, the elongated conformation observed through SAXS-MD leads to a puckered (out of plane) form when observed after a 90° rotation around the *X*-axis, consistent with the breathing motion suggested by the RMSF and sugar pucker analyses. While the models fill the volumetric space in the bead model (Figure 4j), we observe protruding volumetric space for each kissing loop junction which is filled through the models only at chain A:B and D:E junctions. This observation supports our hypothesis that similar stretching via the breathing movement should occur between the kissing loop regions of other opposing chain pairs, i.e., chain B:C and E:F and chain C:D and F:A. To fully explore this coordinated breathing motion, additional modeling using the SAXS-MD multiple replica method would be valuable. This approach, involving multiple replicas with different starting velocities, could help elucidate whether these movements are nucleotide sequence dependent and how they contribute to the overall NANP dynamics.

### SAXS Analysis of Functional RNA NANPs

**Figure 5:**
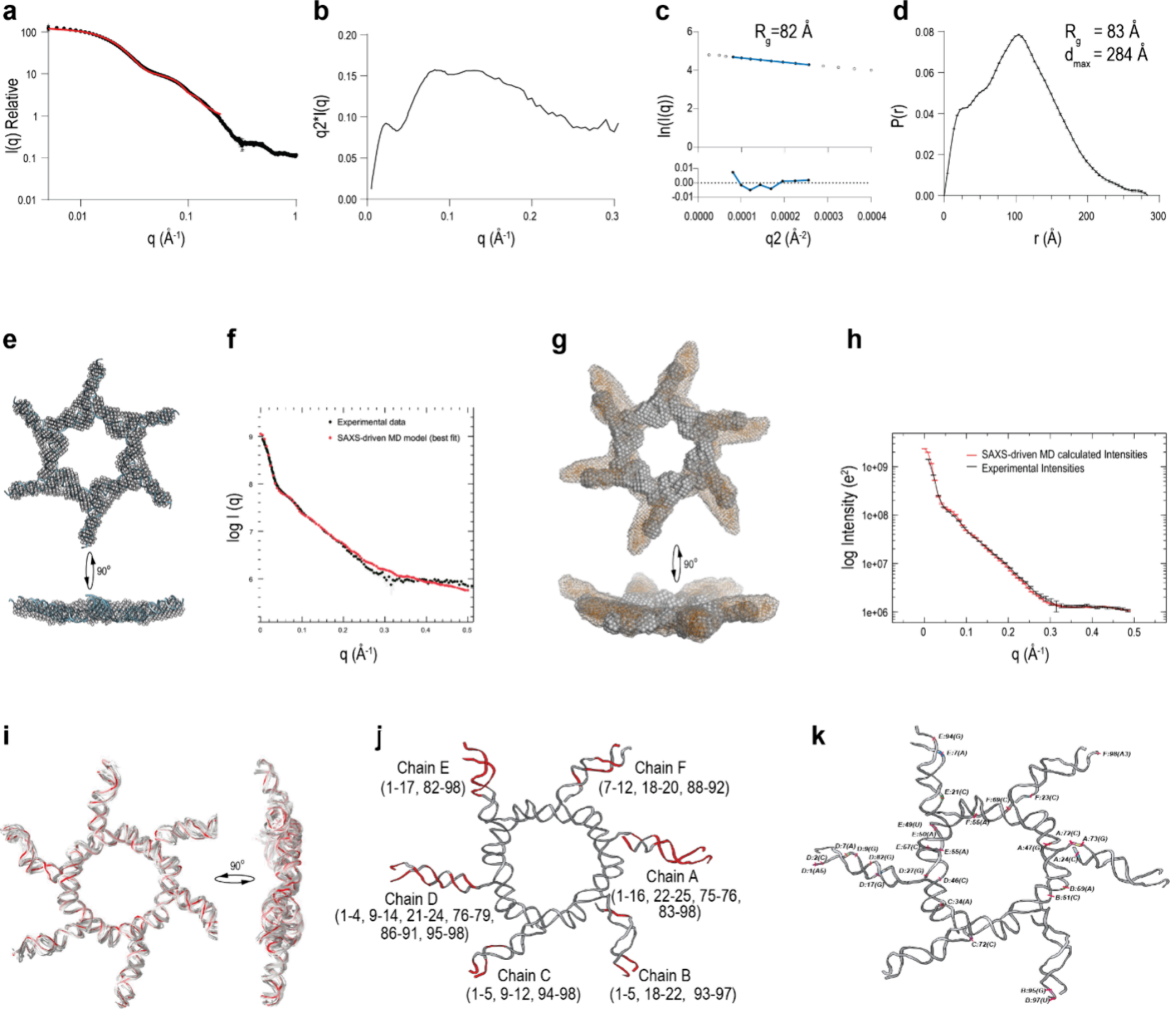
SAXS analysis of the functional RNA NANPs. (a) The experimental SAXS profile of the functional RNA NANPS is shown as black dots with the calculated scattering of the bead model (e) shown as a solid red line. (b) The Kratky plot of the scattering profile. (c) The Guinier fit of the data with the linear fit appearing as a solid blue line. The residuals of the fit are plotted directly underneath the Guinier plot. (d) The pairwise distance distribution of the SAXS data, calculated over 0.005–0.2 Å^–1^. (f) SAXS-MD best fit model obtained from 10 ns simulation fit against the SAXS data using WAXSIS server. (g) The bead model generated using rigid body modeling (SASREF) of the functional RNA monomer to fit the experimental data with P6 symmetry is shown with the anticipated three-dimensional structure superimposed. (h) Average SAXS intensities calculated using the RERUN module of GROMACS-SWAXS (3.5 to 10 ns SAXS-MD trajectory). (i) Top 10 representative models extracted from last 5 ns of 10 ns simulation (gray) with best fit structure in red. (j) SAXS-MD RNA structure model, with key dynamic regions highlighted as per the legend in SI Figure S8B. (k) SAXS-MD RNA structure model highlighting residues showing distinct sugar pucker conformational deviations, with residues displayed in distinct colors as per the legend in SI Figure S8C.

As discussed earlier, the two unpaired nucleotides linking the DS RNA arms to the section forming the body of the RNA NANP (Figure 1b) are anticipated to add the flexibility to the functional RNA NANP compared to the nonfunctional analog, as evident from the Kratky plots of each (Figure 5b vs Figure 4b). The *R*_g_/*R*_h_ ratio of this assembly falls within the range of 0.81–0.82, suggesting behavior akin to a globular particle in solution, albeit not strictly spherical. Despite appearing generally with a *d*_max_ close to or even exceeding 300 Å in some cases in AFM micrographs, the pairwise distance distribution function estimates the *d*_max_ to be 284 Å (Figures 1b and 5d).

It is expected that interaction with the mica surface biases the conformation of the functional RNA NANPs into an elongated and planar configuration. Conformational bias of nucleic acid nanostructures is recognized as an issue in previous AFM measurements.^28^ Previous studies of this structure, using cryo-EM, show a crown-like shape, with the DS RNA arms extended slightly out of the plane in a pinwheel-like shape.^25^

The initial bead model of this particle follows a similar approach to that of the RNA NANP, spanning a q range of 0.005–0.2 Å^–1^ using SASREF to fit the experimental data. The functional RNA monomer bead model is employed, incorporating a 6-fold rotational symmetry constraint, which aligns with the rationale explained for the RNA NANPs. The resulting model confirms the puckering of the DS RNA arms out of the plane, consistent with observations from previous studies. The data for the functional RNA NANPs exhibits minimal error, generally remaining below 1% across the range utilized to generate the rigid body model.

The functional RNA NANPs, with its increased complexity due to the addition of DS sequences, displayed notable dynamic behavior during the SAXS-MD simulation. The trajectory analysis revealed incomplete convergence, with both RMSD and *R*_g_ showing continuous increase even after the waxs-t-target time point, suggesting that the structure continued to explore new conformational states throughout the simulation period (SI Figure S8A). RMSF analysis revealed systematic patterns of flexibility across all six chains (A-F), with several distinct dynamic regions highlighted in red in Figure 5j. Each chain showed characteristic flexible regions viz. Chain A (residues 1–15, 72–76, 93–98), Chain B (residues 15–22, 93–97), Chain C (residues 5, 9–12, 94–98), and similar patterns in Chains D-F (SI Figure S8B). These regions, exhibiting RMSF values above the 75th-percentile threshold, predominantly correspond to the functional arms and their junction points with the core ring structure.

Local conformational analysis through sugar pucker phase angles revealed specific residues with distinct conformational preferences (SI Figure S9C). Notable variations were observed in residues D 27(G), E 49(U), F 69(C), C 27(C), B 51(C) and A 47(G), among others, as shown in Figure 5k. These residues, distributed across different chains and particularly concentrated at the interfaces between the core ring structure and functional arms, display significant deviations from typical A-form RNA conformations. The sugar pucker amplitude distributions further supported these findings, with these residues displaying broader distributions indicative of enhanced conformational flexibility, as evidenced by the distinct peaks and broader distributions in the amplitude plot. The strategic location of these flexible residues, together with their noncanonical conformations, appears to facilitate the characteristic crown-like architecture of the functional NANPs.

The SAXS-MD best fitting model also shows a crown-like shape of the functional RNA NANPs which is puckered in a pinwheel-like shape (Figure 5g). This architecture appears to be stabilized by the coordinated conformational preferences of the junction residues identified in the sugar pucker analysis. As compared to the initial predicted model (Figure 5e), the SAXS-MD model shows a better fit in the low q region, shown in SI Figure S9A. Structurally, the model generated after SAXS-MD shows flexibility in the positions of the six DS RNA arms enabled by the conformational plasticity at the junction residues, contributes to the overall crown-like shape compared to the initial predicted model (SI Figures S9B(i-ii)), as observed with the functional RNA monomer. The single model to data fit represented in Figure 5f vary in the region beyond 0.2 Å^–1^, depicting that no single structure can explain the SAXS data for the flexible RNA NANPs. Hence, the ensemble models generated from the SAXS-MD trajectories fit better the experimental SAXS data as depicted in Figure 5h. Also, the Guinier *R*_g_ calculated from both on the fly curve (SI Figure S9C) as well as average SAXS curve (Figure 5h) is approximately 89 Å, which is slightly larger than the experimental *R*_g_ values. Owing to the large size of this molecule and the computational cost required to perform a longer simulation, only the q region up to 0.5 Å^–1^ was used. The top 10 best fitting models extracted from the SAXS-driven MD trajectory (Figure 5i) show little deviation for the RNA NANP and more flexibility in the DS arms. These models visually align with the bead model generated as described above and shown in Figure 5g.

The distribution of dynamic regions, coupled with the continuous increase in global parameters (RMSD and *R*_g_), suggests that the functional RNA nanoring requires more extensive sampling to fully capture its conformational landscape. The presence of six DS arms significantly increases the system’s complexity, leading to coordinated motions that affect both the core ring structure and the attached DS arms. Future simulations would benefit from significantly longer trajectory times (>50 ns) and the implementation of multiple replica exchange molecular dynamics (REMD) to ensure adequate sampling of all relevant conformational states. The systematic variation in sugar pucker conformations and the persistent structural changes observed indicate that this enhanced sampling would be particularly valuable for understanding the relationship between local flexibility at the DS attachment points and global structural dynamics.

Compared to previous attempts at modeling the RNA NANPs, the approach presented here involves first addressing the structure of the monomers and then assembling the full NANP structure from these units. This approach has proven to be more successful, although there is still opportunity for refinement. The modeling efforts utilizing SAXS-MD have not only enhanced model refinement against SAXS data but have also enabled the identification of regions in the RNA molecule that contribute to their flexibility in solution. However, these analyses reveal that no single model can achieve the ideal χ^2^ due to the inherent flexibility of the RNA molecules. The SAXS-MD trajectories as a collective aid in determining the average shape, which visually resembles the bead models. Given that all RNA molecules exhibit inherent flexibility in solution (as depicted through Kratky plots), it proves more advantageous to conduct SAXS-MD to elucidate the regions contributing to flexibility rather than refining a single model against SAXS data.

## Conclusion

The conventional approaches to characterizing novel NANPs, such as utilizing a combination of EMSA, DLS, and AFM, prove effective in confirming NANPs’ assembly efficiencies and general morphology. However, these techniques offer only a partial view of the structures. Incorporating SAXS in the characterization of NANPs, or any other RNA and DNA molecules with conserved structures, becomes imperative to gain comprehensive insight into their true dimensionalities and shape in the solution environment. For nucleic acid structures that depend on both intra- and intermolecular bonding for full assembly, like the 6- and 12-stranded RNA NANPs studied here, the current approach proves effective. Although further enhancements could be explored through MD simulations encompassing the entire structure and surrounding solvation layers, these methods are computationally and time demanding for larger assemblies. The methodology demonstrated here for assessing the three-dimensional structure of multistranded functional NANPs is transferrable to other systems, particularly those constructed from discrete monomeric units. We showcase that SAXS provides significant structural insights, including information on flexibility and three-dimensional models, and should be a fundamental step in evaluating the structure of nucleic acid nanomaterials alongside DLS and AFM. Importantly, the SAXS-MD modeling approach accommodates flexibility and models high-q data, allowing for the inclusion of finer molecular details to improve the interpretation of the one-dimensional profile. While bead models are useful for elucidating the overall shape of the system, they utilize only a fraction of the available data. We demonstrate that while MD models superimposed with bead models serve as a helpful visual aid, optimizing the fits of MD to experimental intensities and errors represents the optimal method for validating the best-fit models.

By leveraging this methodology, various areas of nucleic acids research can gain insights into the spatial arrangement, intermolecular interactions, and overall conformation of complex structures and nanoassemblies before more detailed experimental characterization. The predictive capability of our approach adds additional value in understanding the behavior and functionality of diverse nanomaterials spanning broad areas of investigations such as RNA and DNA nanotechnology, materials science, and biomedicine.

### Limitations and Future Directions

The present study, while providing valuable insights into structures of RNA NANPs, has certain limitations that offer opportunities for future research. Our analysis primarily relies on relatively short time scale simulations, which may not fully capture the complete range of conformational dynamics suggested by the experimental SAXS data. To address this limitation and more thoroughly investigate the conformational landscape of these constructs and any other NANPs, including rare or transient states that may be functionally relevant, extended simulations or advanced sampling techniques such as multiple replica exchange molecular dynamics (REMD) would be beneficial. These approaches could provide a more extensive exploration of the conformational space along with nucleotide sequence specificity at the kissing loop junctions, potentially revealing additional structural states not observed in our current simulations.

Furthermore, our study hints at potential functional implications based on structural differences, warranting a more focused investigation into structure–function relationships. For example, nonfunctional RNA NANPs differ from DS-RNA functional NANPs in their immunostimulatory properties, as previously reported.^10^ Despite the presence of 5′ triphosphates on both functional and nonfunctional RNA monomers and NANPs, Retinoic acid-inducible gene I (RIG-I) receptors can only be activated by the functional analogs; structural differences between the functional and nonfunctional NANPs may provide insight into why RIG-I presents this selectivity.

This selectivity appears to be influenced by specific conformational features revealed through our sugar pucker analysis. Key junction residues D-27(G), E-49(U), F-69(C), C-27(C), B-51(C), and A-47(G) exhibit distinct conformational preferences that correlate with the observed differences in RIG-I activation. These residues, strategically positioned at chain interfaces, show significant deviations from typical A-form RNA conformations in their sugar pucker phase angles and amplitudes (SI Figure S8C). Their noncanonical conformations appear to contribute to the enhanced exposure of 5′ regions observed in the SAXS-MD models.

The electrostatic distribution on the constructs before and after SAXS-MD simulations was analyzed (SI Figure S10). The analysis of models before and after SAXS-MD reveals increased exposure and solvent accessibility of the functional units 5′ regions when SAXS-MD is applied. The electrostatic charge distribution is also more negative in the case of functional units (SI Figure S10D) compared to nonfunctional ones (SI Figure S10C). This predicted higher exposure may provide a more favorable binding site for RIG-I, which would explain why only functional RNA NANPs can activate RIG-I (SI Figure S10E). The correlation between sugar pucker dynamics at these junction positions and enhanced RIG-I activation suggests that local conformational preferences play a crucial role in determining the global accessibility of immunostimulatory motifs. Future studies could incorporate more structurally versatile structures with assessed biological activities to enhance our understanding of how NANP dynamics provide crucial insights into their functional properties and interactions with other biological materials, paving the way for more precise design and optimization of NANPs intended for biomedical applications.

## Data Availability

The best fitting models, necessary codes for conduction the SAXS-MD simulations and downstream analysis are available at https://zenodo.org/records/14107929

## Supporting Information Available

All sequences used for nonfunctional and functional RNA NANPs, structural information on nonfunctional and functional RNA monomers without Mg^2+^, SAXS-MD models of nonfunctional and functional RNA monomers and RNA NANPs, and SAXS-MD parameters used for different analyzed RNA structures (PDF)
