# Supplementary material for: Small-Angle X-ray Scattering (SAXS) Combined with SAXS-Driven Molecular Dynamics for Structural Analysis of Multistranded RNA Assemblies
Source: ACS Appl Mater Interfaces. 2024 Nov 27;16(49):67178–91. doi: 10.1021/acsami.4c12397 (PMC11637918; doi:10.1021/acsami.4c12397)
Supplement: Supplementary file 1 — am4c12397_si_001.pdf [file am4c12397_si_001.pdf]

Supporting Information

Small-angle X-ray Scattering (SAXS) Combined with SAXS-driven Molecular Dynamics for Structural Analysis of Multistranded RNA Assemblies

Lewis A. Rolband[1#], Kriti Chopra[2#], Leyla Danai[1], Damian Beasock[1], Hubertus J.J. van Dam[3], Joanna K. Krueger[1*], James Byrnes[4*], Kirill A. Afonin[1*]

1 - Nanoscale Science Program, Department of Chemistry, University of North Carolina Charlotte, Charlotte, NC 28223, USA

2 - Computational Science Initiative, Brookhaven National Laboratory, Upton, NY 11973, USA.

3 - Condensed Matter Physics and Materials Science Dept, Brookhaven National Laboratory, Upton, NY 11973, USA.

4 - National Synchrotron Light Source II, Brookhaven National Laboratory, Upton, NY 11973, USA

# - these authors contributed equally to this project.

*Author to whom correspondence should be addressed: kafonin@charlotte.edu, jbyrnes@bnl.gov and Joanna.Krueger@charlotte.edu

Sequences used in this project

RNA NANPs

A: 5'GGGAACCGUCCACUGGUUCCCGCUACGAGAGCCUGCCUCGUAGC

B: 5'GGGAACCGCAGGCUGGUUCCCGCUACGAGAGAACGCCUCGUAGC

C: 5'GGGAACCGCGUUCUGGUUCCCGCUACGAGACGUCUCCUCGUAGC

D: 5'GGGAACCGAGACGUGGUUCCCGCUACGAGUCGUGGUCUCGUAGC

E: 5'GGGAACCACCACGAGGUUCCCGCUACGAGAACCAUCCUCGUAGC

F: 5'GGGAACCGAUGGUUGGUUCCCGCUACGAGAGUGGACCUCGUAGC

Functional RNA NANPs

DS-A:
5'GGGAACCGUCCACUGGUUCCCGCUACGAGAGCCUGCCUCGUAGCUUCGGUGGUGCAGAUGAACUUCAG
GGUCA

DS-B:
5'GGGAACCGCAGGCUGGUUCCCGCUACGAGAGAACGCCUCGUAGCUUCGGUGGUGCAGAUGAACUUCAG
GGUCA

DS-C:
5'GGGAACCGCGUUCUGGUUCCCGCUACGAGACGUCUCCUCGUAGCUUCGGUGGUGCAGAUGAACUUCAG
GGUCA

DS-D:
5'GGGAACCGAGACGUGGUUCCCGCUACGAGUCGUGGUCUCGUAGCUUCGGUGGUGCAGAUGAACUUCAG
GGUCA

DS-E:
5'GGGAACCACCACGAGGUUCCCGCUACGAGAACCAUCCUCGUAGCUUCGGUGGUGCAGAUGAACUUCAG
GGUCA

DS-F:
5'GGGAACCGAUGGUUGGUUCCCGCUACGAGAGUGGACCUCGUAGCUUCGGUGGUGCAGAUGAACUUCAG
GGUCA

Strand complementary to DS-A – DS-F:

5'pACCCUGAAGUUCAUCUGCACCACCG

Supporting Figures

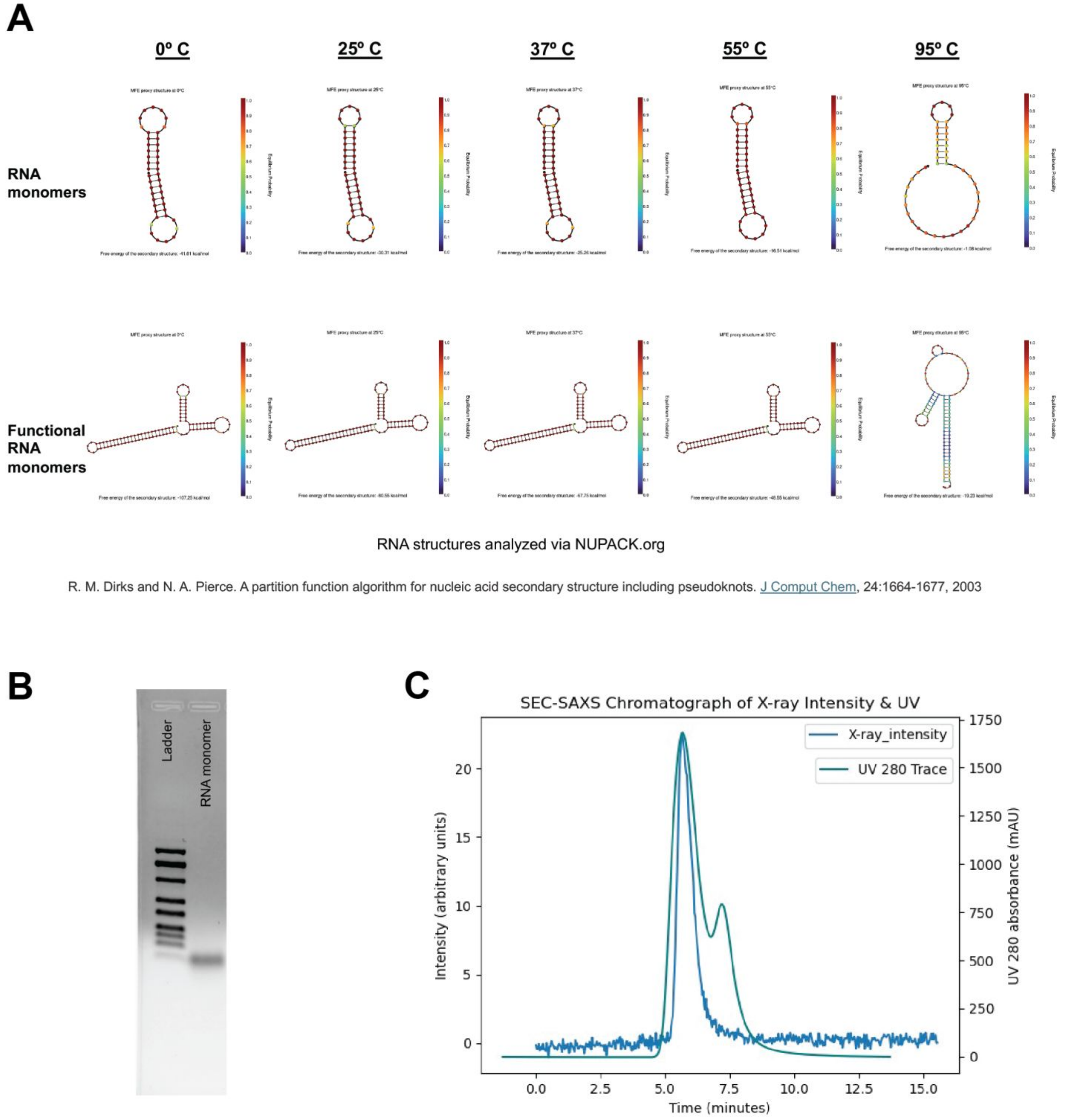

Fig S1. Analysis of the non-functional and functional RNA monomers. (**A**) Secondary structures predicted using NUPACK[1] for non-functional and functional RNA monomers at a range of temperatures. (**B**) 1.5% agarose EMSA of non-functional RNA monomer prepared in buffer without Mg^{2+}. (**C**) SEC-SAXS results of the non-functional RNA monomer run without Mg^{2+}. Elution profile shows a tailing shoulder in both the SAXS (solid blue) and UV (solid green) traces. Use of SEC-SAXS at LIX beamline allows for the simultaneous collection of X-ray and UV data. UV offset in time is adjusted by a scaling factor to align peaks.

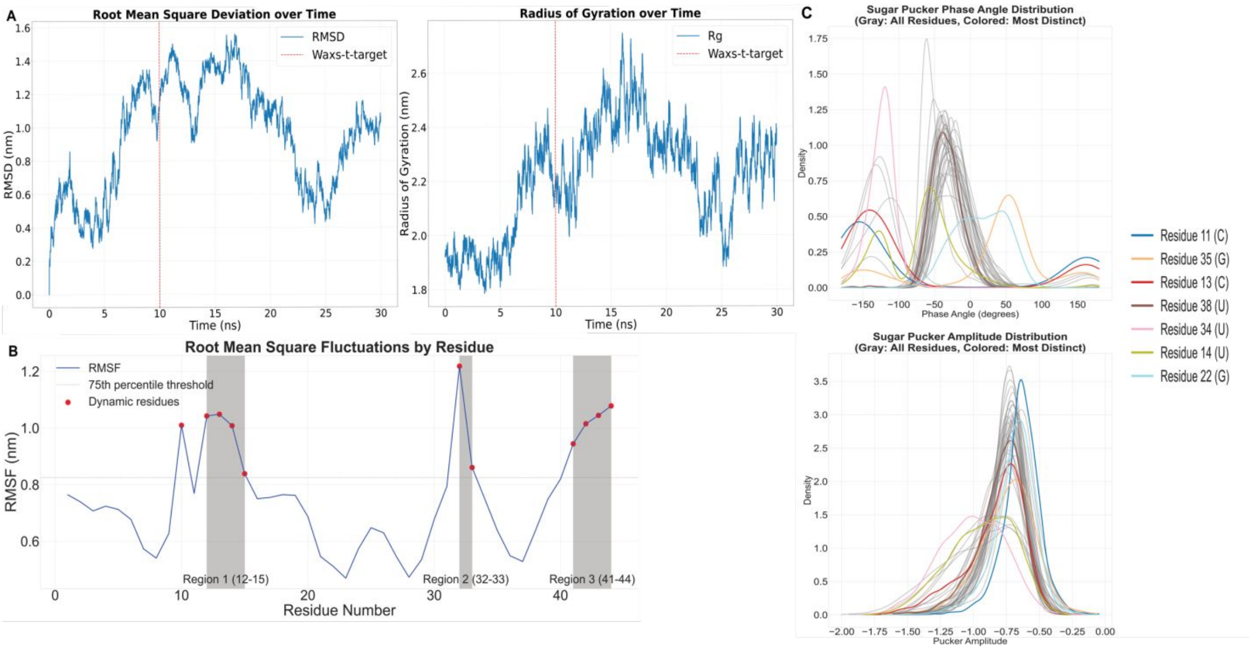

Fig S2 SAXS-driven MD trajectory analysis of RNA non-functional monomer: (**A**) Root Mean Square Deviation (RMSD) and Radius of Gyration (Rg) over time for the 30 ns SAXS-MD simulation. Waxs-t-target represents the time until which the SAXS data was incorporated in the simulation. The time beyond waxs-t-target represents the simulation under the effect of forcefields as well as SAXS experimental data. (**B**) Root Mean Square Fluctuations (RMSF) by Residue, the red dots denote dynamic residues and 2 or more than 2 consecutive residues are defined as dynamic regions i.e. Region 1 (12-15), Region 2 (32-33) and Region 3 (41-44). (**C**) Sugar Pucker Angle and Amplitude density plots generated from SAXS-MD trajectories. The highlighted residues showed deviation more than 2 sigma and hence are considered to be contributing to dynamics and flexibility of the RNA non functional monomer model.

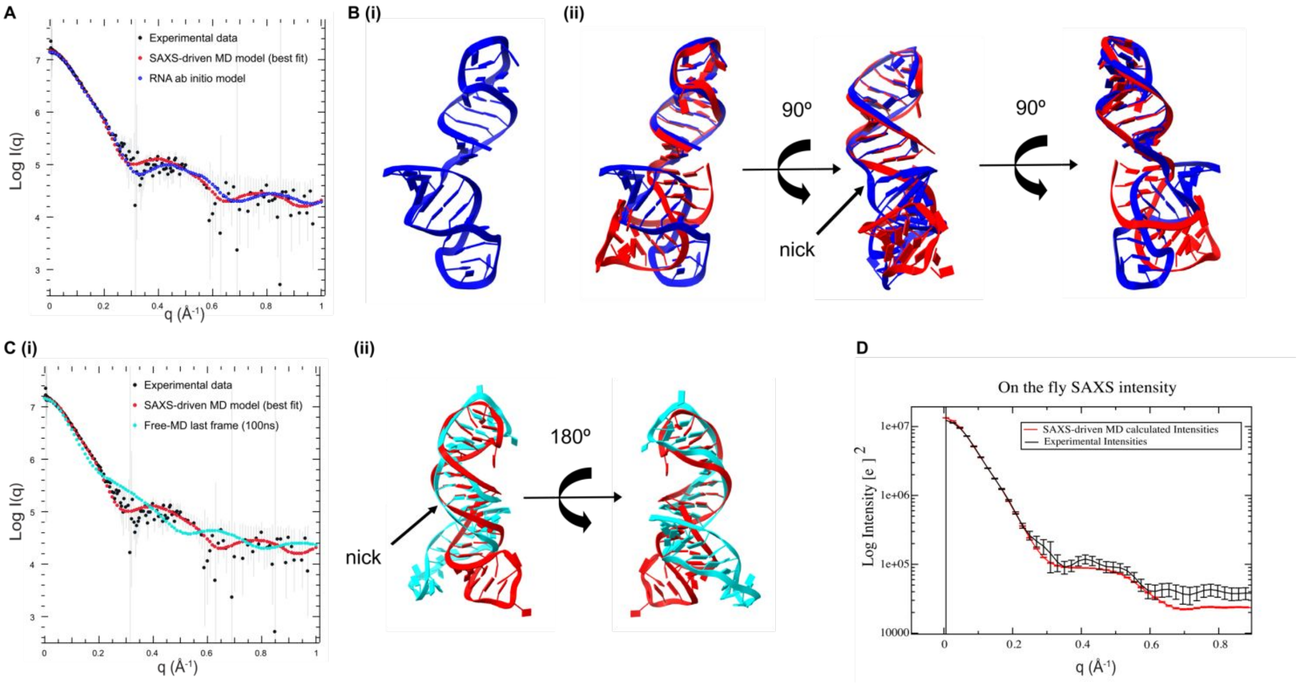

Fig S3. SAXS analysis of the non-functional RNA monomers. (**A**) Model to data fit comparison of RNA predicted model with SAXS-MD model using WAXSIS server. (**B**) (i) RNA predicted model (ii) Superimposition of RNA predicted model onto SAXS-MD model generated using RNAalign (Gong et al 2019). The nick (shown by the arrow) in the RNA structure obtained from the predicted model (blue) is resolved during the SAXS-driven MD (red). (**C**) (i) Model to data fit comparison of SAXS-MD model with extended free-MD of 100ns depicts that a longer unconstrained MD cannot generate a model that fits the data as well as a shorter SAXS- the predicted model is resolved via the SAXS-MD simulation. (ii) Superimposition of free-MD 100ns frame (cyan) on SAXS-MD best model (red) depicts the kink in the *ab intio* model and is resolved via the SAXS-MD simulation. (**D**) On the fly curve generated from 30 ns SAXS-MD model.

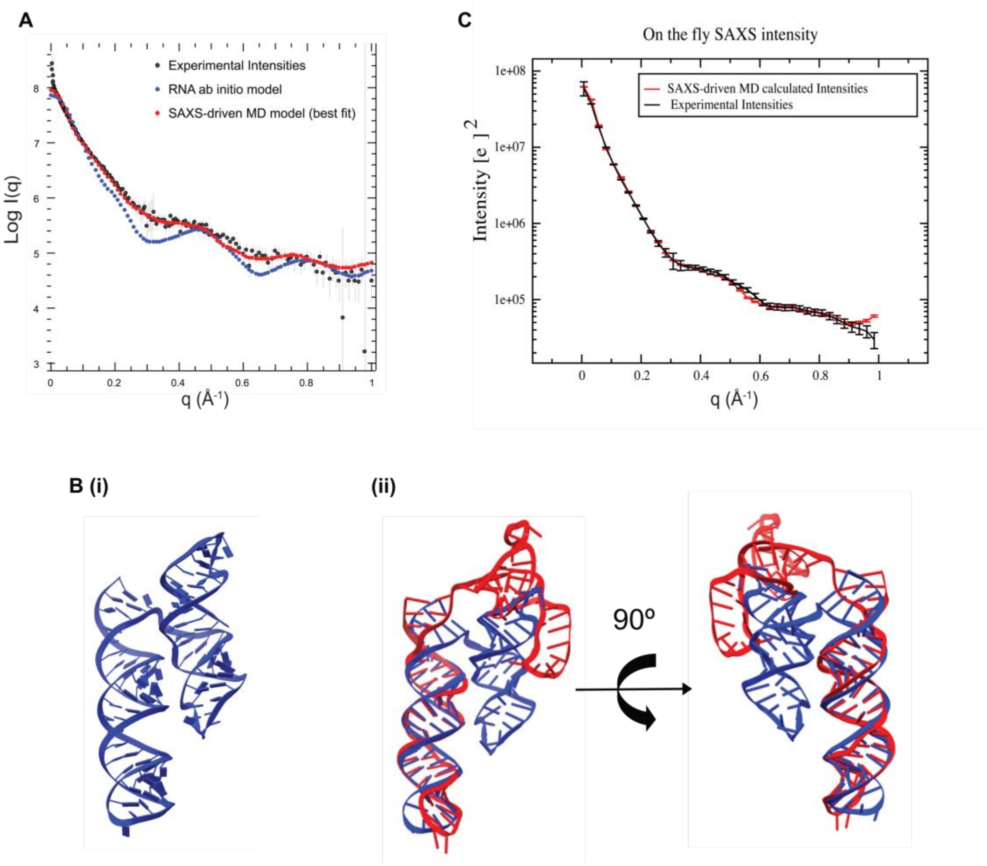

Fig S4. **SAXS analysis of the functional RNA monomers.** (**A**) Model to data fit comparison of RNA predicted model with SAXS-MD model using WAXSIS server. (**B**) (i) RNA predicted model (ii) Superimposition of RNA predicted model onto SAXS-MD model generated using RNAalign (Gong et al 2019). (**C**) On the fly curve generated from 15 ns SAXS-MD simulation.

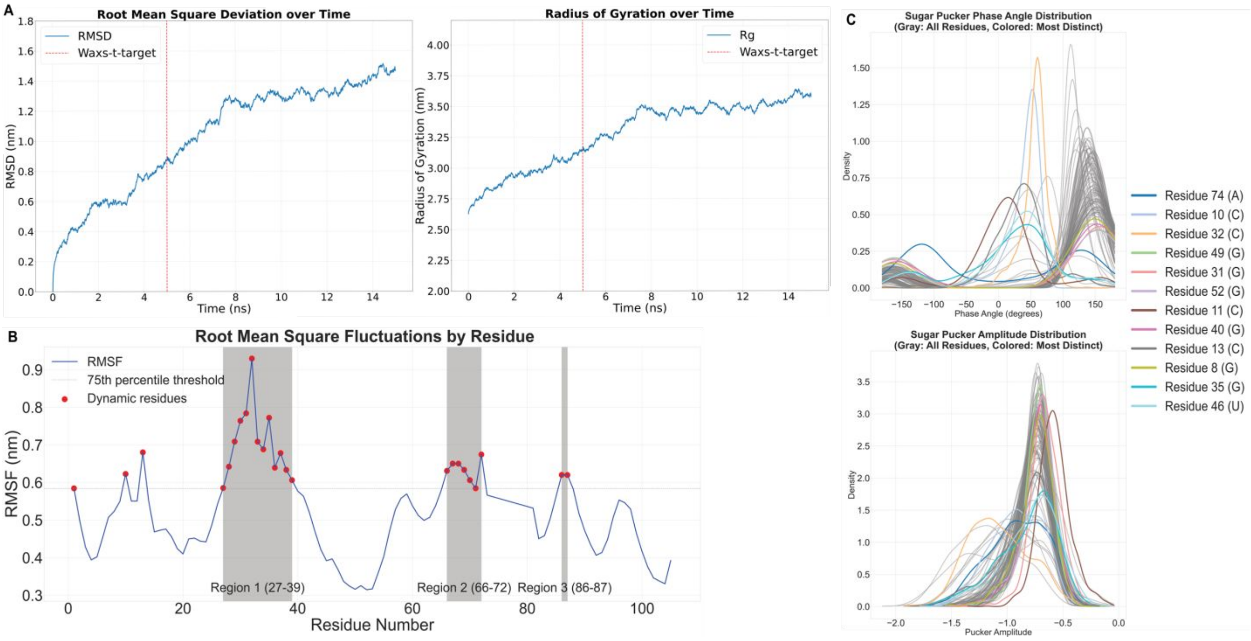

Fig S5 SAXS-driven MD trajectory analysis for RNA functional monomer: (**A**) Root Mean Square Deviation (RMSD) and Radius of Gyration (R_g) over time for the 15 ns SAXS-MD simulation. Waxs-t-target represents the time until which the SAXS data was incorporated in the simulation. The time beyond waxs-t-target represent the simulation under the effect of forcefields as well as SAXS experimental data. (**B**) Root Mean Square Fluctuations (RMSF) by Residue, the red dots denote dynamic residues and 2 or more than 2 consecutive residues are defined as dynamic regions i.e. Region 1 (27-39), Region 2 (66-72) and Region 3 (86-87). (**C**) Sugar Pucker Angle and Amplitude density plots generated from SAXS-MD trajectories. The highlighted residues showed deviation of more than 2 sigma and hence are contributing to dynamics and flexibility of the RNA functional monomer model.

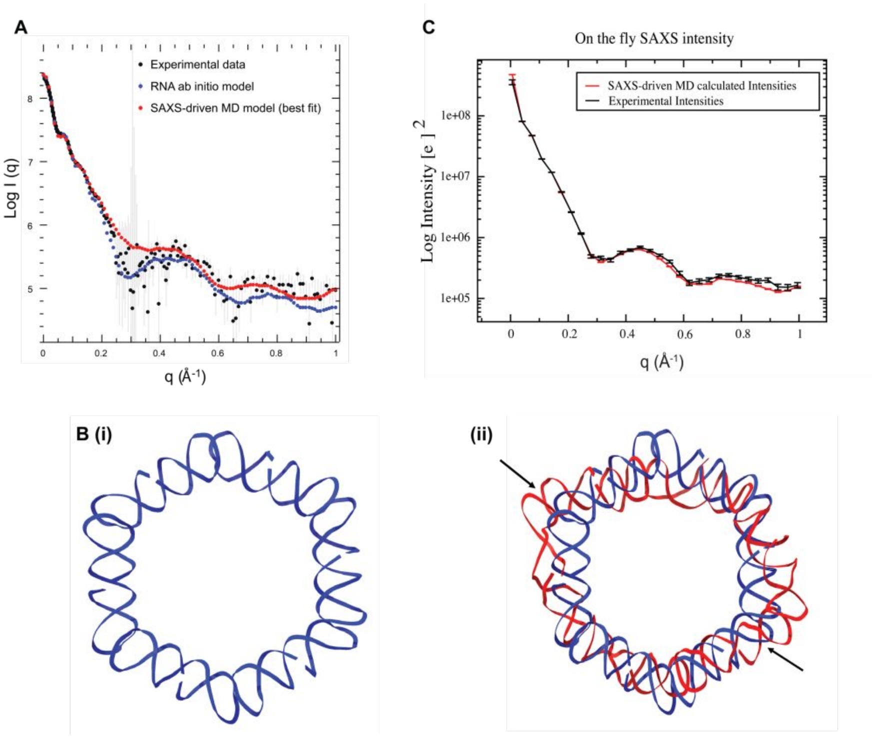

Fig S6. SAXS analysis of the non-functional RNA NANPs. (**A**) (i) Model to data fit comparison of RNA predicted model with SAXS-MD model using WAXSIS server. (**B**) (ii) RNA predicted model (iii) Superimposition of RNA predicted model onto SAXS-MD model extracted from SAXS-MD trajectory. The arrows depict the stretching of ring with opposing forces at the kissing loop junctions. (**C**) On the fly curve generated from 12 ns SAXS-MD.

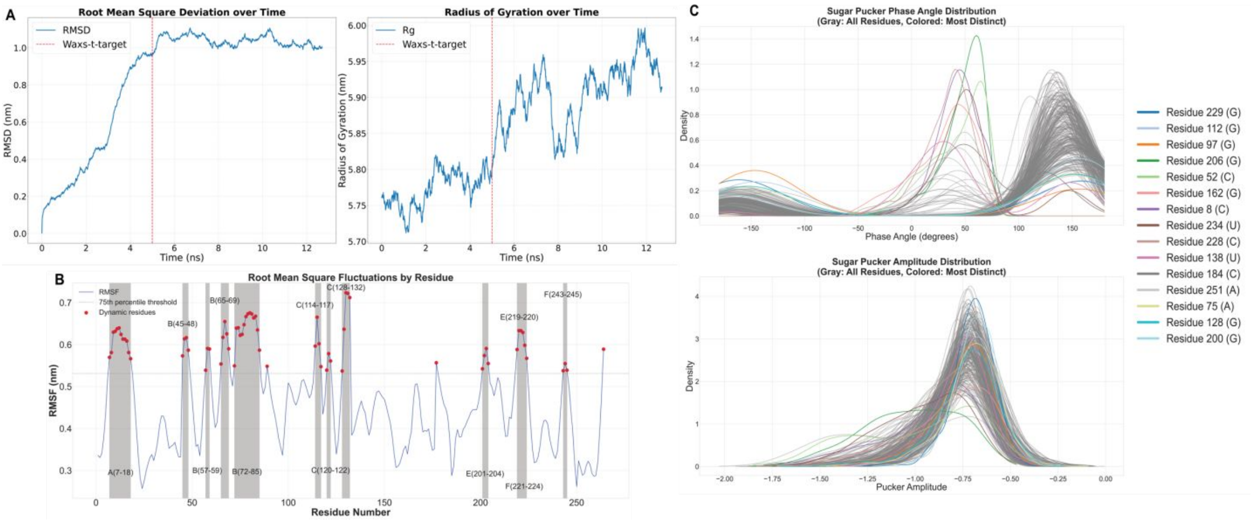

Fig S7. SAXS-driven MD trajectory analysis for RNA non-functional ring: (**A**) Root Mean Square Deviation (RMSD) and Radius of Gyration (R_g) over time for the 12 ns SAXS-MD simulation. Waxs-t-target represents the time until which the SAXS data was incorporated in the simulation. The time beyond waxs-t-target represent the simulation under the effect of forcefields as well as SAXS experimental data. (**B**) Root Mean Square Fluctuations (RMSF) by Residue, the red dots denote dynamic residues and 2 or more than 2 consecutive residues are defined as dynamic regions). (**C**) Sugar Pucker Angle and Amplitude density plots generated from SAXS-MD trajectories. The highlighted residues showed deviation of more than 2 sigma and hence are contributing to dynamics and flexibility of the RNA non-functional ring model.

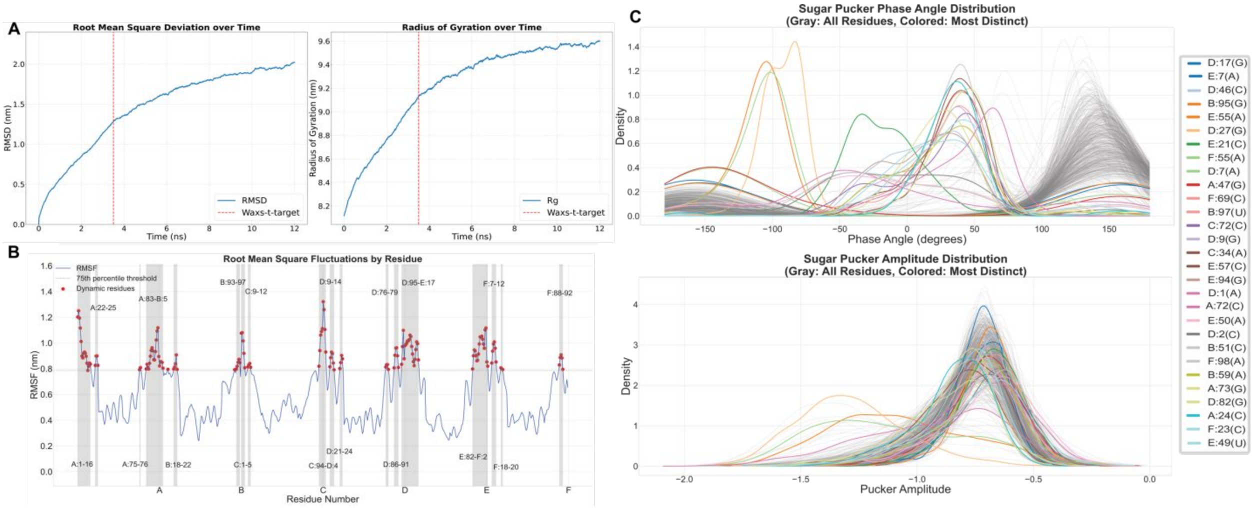

Fig S8. SAXS-driven MD trajectory analysis for RNA functional ring: (**A**) Root Mean Square Deviation (RMSD) and Radius of Gyration (R_g) over time for the 12 ns SAXS-MD simulation. Waxs-t-target represents the time until which the SAXS data was incorporated in the simulation. The time beyond waxs-t-target represent the simulation under the effect of forcefields as well as SAXS experimental data. (**B**) Root Mean Square Fluctuations (RMSF) by Residue, the red dots denote dynamic residues and 2 or more than 2 consecutive residues are defined as dynamic regions). (**C**) Sugar Pucker Angle and Amplitude density plots generated from SAXS-MD trajectories. The highlighted residues showed deviation of more than 2 sigma and hence are contributing to dynamics and flexibility of the RNA functional ring model.

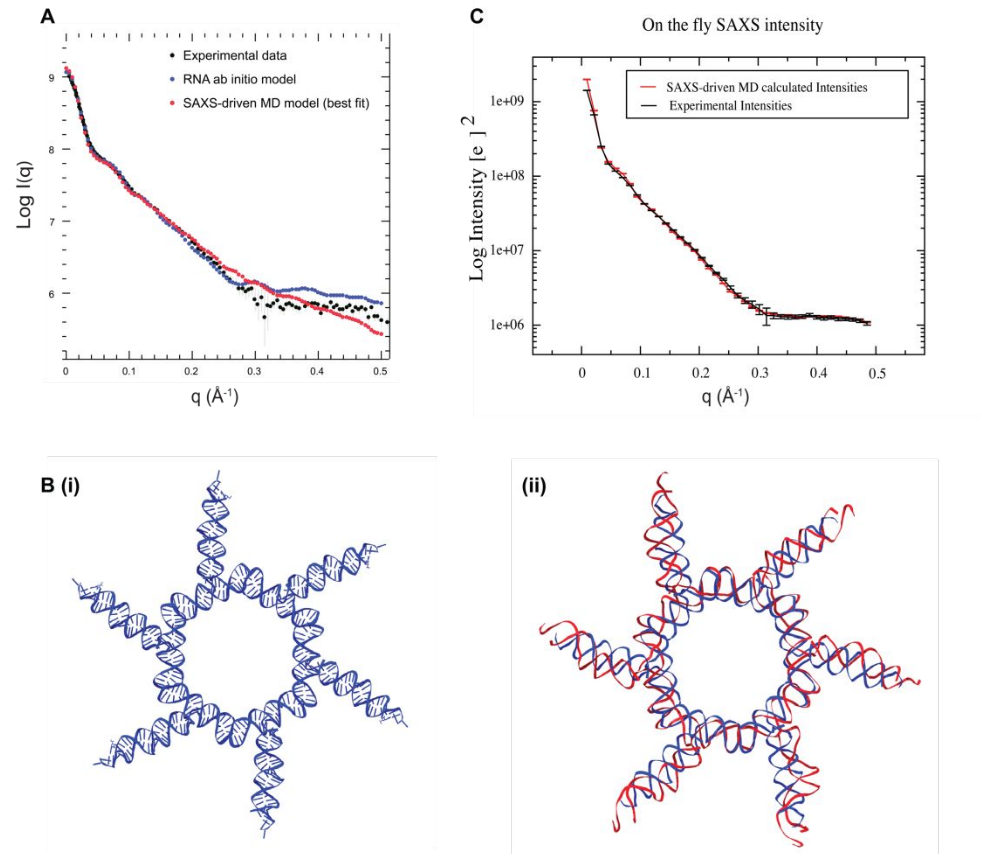

Fig S9. SAXS analysis of the functional RNA NANPs (**A**) Model to data fit comparison of RNA predicted model with SAXS-MD model using WAXSIS server. (**B**) (i) RNA predicted model (ii) Superimposition of RNA predicted model onto SAXS-MD model extracted from SAXS-MD trajectory. (**C**) On the fly curve generated from 12 ns SAXS-MD simulation.

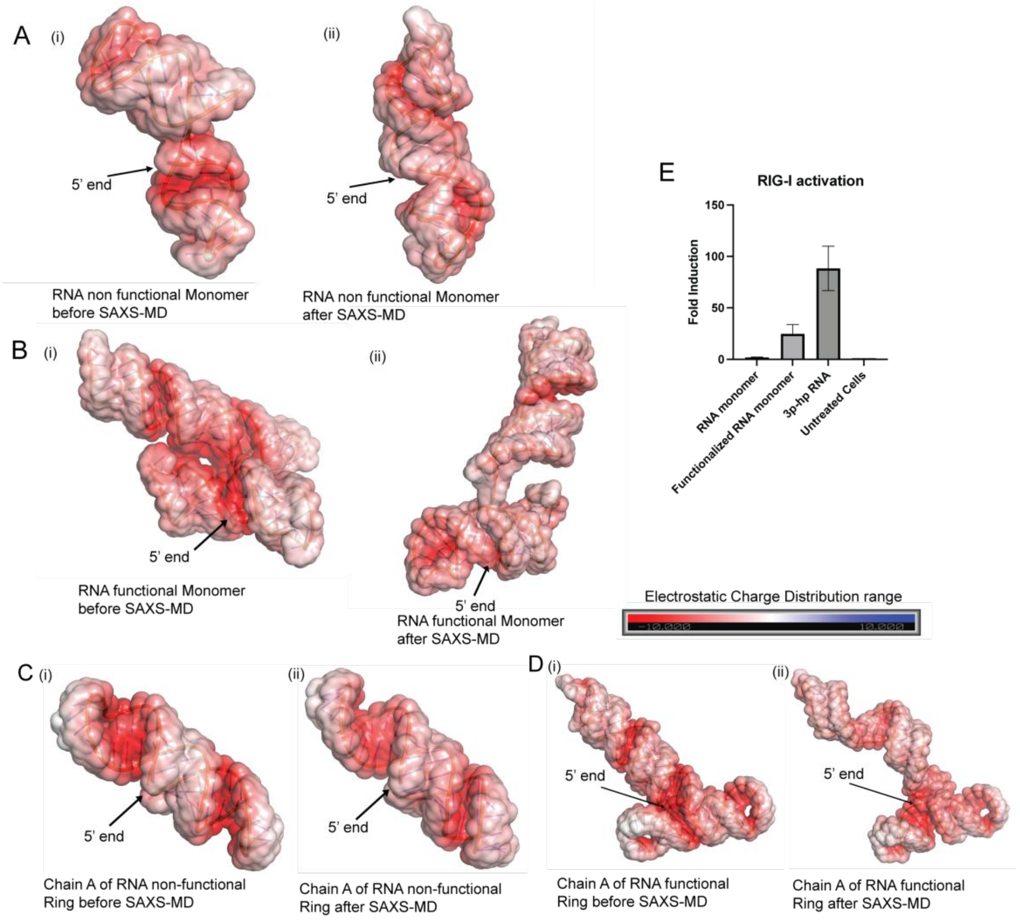

Fig S10. (**A**) Electrostatic distribution on the non-functional RNA monomer (i) before (ii) and after SAXS-MD simulation (**B**) Electrostatic distribution on chain A of functional RNA monomer (i) before (ii) and after SAXS-MD simulation (**C**) Electrostatic distribution on chain A of the non-functional ring (i) before (ii) and after SAXS-MD simulation (**D**) Electrostatic distribution on chain A of functional ring (i) before (ii) and after SAXS-MD simulation (**E**) RIG-I activation of RNA monomer, functionalized RNA monomer, and 5' triphosphate RNA (3p-hp) as compared to untreated HEK Lucia RIG-I cells. All treatments were at a final concentration of 10 nM. The average of 3 biological repeats are plotted ± SEM.

Supporting Table

Table S1. SAXS-MD parameters used for different analyzed structures.

Parameters/Models	RNA monomers	Functional RNA monomers	RNA NANPs	Functional RNA NANPs
Waxs-t-target	10 ns	5 ns	5 ns	3.5 ns
Waxs-fc	1	1	1	1
Number of q points (waxs-nq)	50	40	30	40
Waxs-startq (nm^{-1})	0.06	0.07	0.065	0.09
Waxs-endq (nm^{-1})	10	9.85	10	4.85
Waxs-solvdens-uncert	0.001 (0.1%)	0.001 (0.1%)	0.001 (0.1%)	0.01 (1%)
Waxs-tau	250 ps	250 ps	250 ps	250 ps
Total simulation time (SAXS-MD)	30 ns	15 ns	12 ns	12 ns

References:

(1) Zadeh, J. N.; Steenberg, C. D.; Bois, J. S.; Wolfe, B. R.; Pierce, M. B.; Khan, A. R.; Dirks, R. M.; Pierce, N. A. NUPACK: Analysis and design of nucleic acid systems. *Journal of Computational Chemistry* **2011**, *32* (1), 170-173. DOI: 10.1002/jcc.21596.
